# Supplementary material for: Are better existing WASH practices in urban slums associated with a lower long-term risk of severe cholera? A prospective cohort study with 4 years of follow-up in Mirpur, Bangladesh
Source: BMJ Open. 2022 Sep 21;12(9):e060858. doi: 10.1136/bmjopen-2022-060858 (PMC9494564; doi:10.1136/bmjopen-2022-060858)
Supplement: Author's manuscript [file bmjopen-2022-060858.draft_revisions.pdf]

BMJ Open

BMJ Open is committed to open peer review. As part of this commitment we make the peer review history of every article we publish publicly available.

When an article is published we post the peer reviewers' comments and the authors' responses online. We also post the versions of the paper that were used during peer review. These are the versions that the peer review comments apply to.

The versions of the paper that follow are the versions that were submitted during the peer review process. They are not the versions of record or the final published versions. They should not be cited or distributed as the published version of this manuscript.

BMJ Open is an open access journal and the full, final, typeset and author-corrected version of record of the manuscript is available on our site with no access controls, subscription charges or pay-per-view fees (http://bmjopen.bmj.com).

If you have any questions on BMJ Open's open peer review process please email
info.bmjopen@bmj.com

BMJ Open

Addressing endemic cholera with improved WASH in urban slums: a prospective cohort study

Journal:	*BMJ Open*
Manuscript ID	bmjopen-2022-060858
Article Type:	Original research
Date Submitted by the Author:	24-Feb-2022
Complete List of Authors:	Kang, Sophie; International Vaccine Institute, Epidemiology, Public Health, and Impact Unit<br>Chowdhury, Fahima; International Centre for Diarrhoeal Disease Research Bangladesh; Griffith University Menzies Health Institute Queensland<br>Park, Juyeon; International Vaccine Institute, Epidemiology, Public Health, and Impact Unit; University of Cambridge, Department of Medicine<br>Ahmed, Tasnuva; International Centre for Diarrhoeal Disease Research Bangladesh<br>Tadesse, Birkneh Tilahun; International Vaccine Institute, Epidemiology, Public Health, and Impact Unit<br>Islam, Md. Taufiqul; International Centre for Diarrhoeal Disease Research Bangladesh<br>Kim, Deok Ryun; International Vaccine Institute, Epidemiology, Public Health, and Impact Unit<br>Im, Justin; International Vaccine Institute<br>Aziz, Asma; International Vaccine Institute, Epidemiology, Public Health, and Impact Unit<br>Hoque, Masuma; International Centre for Diarrhoeal Disease Research Bangladesh<br>Pak, GiDeok; International Vaccine Institute<br>Khanam, Farhana; International Centre for Diarrhoeal Disease Research Bangladesh<br>Ahmmed, Faisal; International Centre for Diarrhoeal Disease Research Bangladesh<br>Liu , Xinxue ; University of Oxford Oxford Vaccine Group<br>Zaman, K; International Centre for Diarrhoeal Disease Research Bangladesh<br>Khan, Ashraful Islam; International Centre for Diarrhoeal Disease Research<br>Kim, Jerome ; International Vaccine Institute<br>Marks, Florian ; International Vaccine Institute; University of Cambridge, Department of Medicine<br>Qadri, Firdausi ; International Centre for Diarrhoeal Disease Research Bangladesh<br>Clemens, John; International Vaccine Institute, Epidemiology, Public Health, and Impact Unit; International Centre for Diarrhoeal Disease Research Bangladesh
Keywords:	EPIDEMIOLOGY, Epidemiology < INFECTIOUS DISEASES, INFECTIOUS

	DISEASES, PUBLIC HEALTH

SCHOLARONE™
Manuscripts

I, the Submitting Author has the right to grant and does grant on behalf of all authors of the Work (as defined in the below author licence), an exclusive licence and/or a non-exclusive licence for contributions from authors who are: i) UK Crown employees; ii) where BMJ has agreed a CC-BY licence shall apply, and/or iii) in accordance with the terms applicable for US Federal Government officers or employees acting as part of their official duties; on a worldwide, perpetual, irrevocable, royalty-free basis to BMJ Publishing Group Ltd ("BMJ") its licensees and where the relevant Journal is co-owned by BMJ to the co-owners of the Journal, to publish the Work in this journal and any other BMJ products and to exploit all rights, as set out in our licence.

The Submitting Author accepts and understands that any supply made under these terms is made by BMJ to the Submitting Author unless you are acting as an employee on behalf of your employer or a postgraduate student of an affiliated institution which is paying any applicable article publishing charge ("APC") for Open Access articles. Where the Submitting Author wishes to make the Work available on an Open Access basis (and intends to pay the relevant APC), the terms of reuse of such Open Access shall be governed by a Creative Commons licence – details of these licences and which Creative Commons licence will apply to this Work are set out in our licence referred to above.

Other than as permitted in any relevant BMJ Author's Self Archiving Policies, I confirm this Work has not been accepted for publication elsewhere, is not being considered for publication elsewhere and does not duplicate material already published. I confirm all authors consent to publication of this Work and authorise the granting of this licence.

For peer review only

Addressing endemic cholera with improved WASH in urban slums: a prospective cohort study

Sophie Kang[1*], Fahima Chowdhury[2,7*], Juyeon Park[1,4*], Tasnuva Ahmed[2], Birkneh Tilahun Tadesse[1], Md. Taufiqul Islam[2], Deok Ryun Kim[1], Justin Im[1], Asma Binte Aziz[1], Masuma Hoque[2], Gideok Pak[1], Farhana Khanam[2], Faisal Ahmmed[2], Xinxue Liu[3], Khalequ Zaman[2], Ashraful Islam Khan[2], Jerome H. Kim[1], Florian Marks[1,4,5], Firdausi Qadri[2], John D. Clemens[1,2,6]

1 International Vaccine Institute, Seoul, Republic of Korea

2 International Centre for Diarrheal Disease Research, Bangladesh, Dhaka, Bangladesh

3 Oxford Vaccine Group, Department of Pediatrics, University of Oxford, Oxford OX3 9DU, UK

4 Department of Medicine, University of Cambridge, Cambridge, United Kingdom

5 University of Antananarivo, Antananarivo, Madagascar

6 UCLA Fielding School of Public Health, Los Angeles, CA 90095-1772, USA

7 Menzies Health Institute Queensland, Griffith University, Gold Coast, Australia

* Contributed equally

Correspondence to Sophie Kang, International Vaccine Institute; Sophie.kang@ivi.int

For peer review only

Abstract

Objectives

To investigate the association between existing household WASH practices and severe cholera risk in a dense urban slum where cholera is highly endemic.

Design, Setting, and Participants

We assembled a large prospective cohort within a cluster randomized trial evaluating the effectiveness of oral cholera vaccine. Our dynamic cohort population (n=193,576) was composed of individuals living in the "non-intervention" clusters of the trial, and were followed over four years. This study was conducted in a dense urban slum community of Dhaka, Bangladesh and cholera surveillance was active in twelve hospitals serving the study area.

Primary outcome measure

First severe cholera episode detected during follow-up period.

Results

The "Better" WASH decision tree found that water quality and access were the most significant factors associated with severe cholera risk. Members of "Better" WASH households, constituting one third of the total population, had a 47% reduced risk of severe cholera (95% CI: 29-69; p-value <0.001), after adjusting for covariates. The protective association between living in a "Better" WASH household and severe cholera persisted in all age groups.

Conclusions

These findings suggest that salutary WASH practices can significantly reduce long-term risk of severe cholera even in highly endemic areas, and future interventions should look to these culturally acceptable WASH practices when designing sustainable cholera programs.

For peer review only

Strengths and limitations of this study

- We followed the cohort under study for four years, making this analysis one of the longest uninterrupted evaluations of the relationship between household WASH and endemic cholera.
- The multivariable WASH prediction rule was validated using a separate sub-population and thus avoided overfitting to the training set.
- Household WASH factors were only evaluated once, either at baseline or when participants entered the study area, and this status applied to the entire follow-up period
- The household WASH variables included in this study were collected in the context of an OCV trial and not optimized for describing WASH factors independently

For peer review only

Introduction

The developing world has seen a rapid expansion of urban areas due, in part, to influxes into squalid urban slums. With an estimated 55% of the world's population living in urban areas, 1-in-3 of those urban dwellers live in slum households. [1] Slum households are defined as those which lack one or more of the following conditions: access to improved water, access to improved sanitation, sufficient living area, and durability of housing.

For peer review only

In Bangladesh urban dwellers currently account for 38% of the population and are expected to exceed 50% by 2030; furthermore, 47% of the urban population lives in slums, where residents are at increased risk for waterborne diseases, including cholera[2,3].

Cholera is a major cause of morbidity and mortality in Bangladesh.[4] Previously thought to be a rural disease in Bangladesh, cholera is now becoming a disease of cities and slums where living conditions create different challenges for cholera control from those encountered in rural settings. [5] Lessons learned from rural areas, and particularly in epidemic situations, may not be applicable to the changing pattern of cholera endemicity in urban areas. Specific studies on how endemic cholera can be controlled in these urban slums are needed.

Exacerbating the urban cholera situation further is the role of climate change. Rising temperatures and increased precipitation associated with climate change are significant predictors of cholera incidence, with strong evidence in studies of Bangladesh where warmer, wetter conditions are associated with major cholera outbreaks. [6] Climate change effects in urban areas have added negative implications for water quality, and studies have found that increased rainfall intensity combined with impervious urban surfaces are significant predictors of combined sewer overflows that greatly impact water quality. [7,8]

Improvements of water quality, sanitation, and hygiene (WASH) and oral cholera vaccine (OCV) are the major tools for the prevention of endemic cholera, including in urban slums. However, while WASH interventions are frequently employed to control cholera, evidence regarding their effectiveness is inconsistent and successful implementation may be stymied by limited cultural acceptability, low uptake, and poor community acceptance. [9, 10, 11, 12] Our cluster, randomized trial (CRT) of OCV and WASH in an urban Dhaka slum, which failed to demonstrate that WASH added to protection against severe cholera by OCV, is illustrative. [13]

In this re-analysis of the CRT, we examine households in the 'non-intervention' arm to investigate whether there were already WASH practices in the slums that correlated with a lower risk of severe cholera and that might contribute to the development of future effective, acceptable, and sustainable WASH interventions in cholera-endemic urban populations.

Methods

Trial and Population

For peer review only - http://bmjopen.bmj.com/site/about/guidelines.xhtml

In 2011, the International Centre for Diarrheal Disease Research, Bangladesh (icddr,b) conducted a cluster randomized control trial entitled ‘Introduction of cholera vaccine in Bangladesh (ICVB)’[13] in six selected wards of Mirpur, Dhaka to evaluate the feasibility and effectiveness of oral cholera vaccine (OCV), both deployed alone and in conjunction with WASH interventions. Vaccination with Shanchol, a two-dose OCV was carried out between 17 February and 6 April, 2011.

Households were grouped into 90 geographic clusters with an average population of 2988 households per cluster (ranging from 2288 to 4299 households per cluster). Clusters were randomly assigned (1:1:1) to one of three arms: a two-dose regimen of OCV alone, OCV with a WASH intervention, or no intervention (control). [13] The two doses of OCV were administered at a 14-day interval. The WASH intervention consisted of a behavioral change intervention which included a household handwashing station featuring a bottle of soapy water and a packet of soap, and a chlorine dispenser for the treatment of household drinking water. [13] Trained community health workers demonstrated handwashing and treatment of drinking water. Healthy, non-pregnant individuals aged one year or older were eligible for vaccination in this cluster randomized trial (CRT), and each cluster was separated from the adjoining cluster by at least a 30-meter buffer area.

Demographic Surveillance

A baseline demographic census was conducted before the start of the ICVB vaccination campaign and updated bi-annually with information on births, deaths, and migrations. Verbal consent for participation in the surveillance was obtained and documented in a questionnaire at the time of the baseline census and at each bi-annual update. A “household” was defined as persons sharing the same cooking pot.

In addition to the basic demographic information, household-level socioeconomic status, water-sanitation-hygiene (WASH) data, and geographical locations of each household were collected during the baseline census. For households that were not present at the baseline census, WASH characteristics were assessed at the first bi-annual census update they were captured in. Household WASH characteristics were not re-assessed for new births in a household already characterized during the study period. All individuals living in the study area were provided with a study identification card containing a unique participant identification (PID) number which was recorded in computerized study databases.

Disease Surveillance

Disease surveillance for cholera was conducted between April 2011 to November 2015 at twelve hospitals serving the study area. Study surveillance staff were present at each health facility throughout the day to facilitate reporting of diarrheal cases from the study area.

Patients from the study area were identified in the treatment centers with their study identification card or by searching their identities in on-site computerized census database.

For peer review only - http://bmjopen.bmj.com/site/about/guidelines.xhtml

Clinical examination was carried out by physicians, and designated study staff completed data forms and obtained fecal specimens after obtaining written informed consent.

A diarrheal visit was defined as having 3 or more loose stools or, 1-2 or an indeterminate number of loose stools with evidence of dehydration, in the 24 hours before presentation[13]. If the date of discharge from an earlier diarrheal visit and the date of symptom onset for the subsequent diarrheal visit were within 7 days of one another, then both visits were considered part of the same diarrheal episode. The onset of a diarrheal episode was defined as starting on the day the patient first reported loose or liquid stools.

Fecal samples were examined for *V. cholerae* O1 or O139 serogroups and Inaba and Ogawa serotypes using conventional methods.[13] A cholera episode was defined as a diarrheal episode in which a fecal specimen yielded *V. cholerae* O1 or O139, with no passage of bloody stools during the episode. Severely dehydrating cholera was defined by the presence of at least two of the following symptoms of severe dehydration: sunken eyes, dry tongue, thirst, irritable condition, less active than usual along with inability to drink, skin pinch goes back slowly, or low volume of radial pulse[13]. A severe cholera episode was one in which the patient exhibited severe dehydration during any visit of the episode. The primary outcome in this analysis was the first severe cholera episode detected during follow-up.

<u>Patient and Public Involvement</u>

This analysis utilizes data that originates from the ICVB cluster randomized trial conducted by the icddr,b in 2011. Given that ten years have passed since the original study, the participants were not directly involved in developing or informing the design of the analysis described in this paper. That said, the original ICVB trial involved strong social mobilization and community engagement to improve the conduct of the study.

The research questions addressed in the ICVB cluster randomized trial were developed due to the pressing need to understand the impact of oral cholera vaccines in urban Bangladesh. Advocacy meetings with local government representatives, pediatric associations, and NGOs were held in order to inform the design and conduct of the ICVB trial.

Analytical Approach

Population Under Follow-up

We considered a dynamic population for this analysis, which included the population present in the non-intervention arm at baseline and new entrants into the non-intervention study area during the study period. For those present at baseline, the start of follow-up was defined as the median date of first Shanchol dose in the nearest intervention cluster. For new residents, the start of follow up was defined as either the date of birth or the date of migration into the study area. The end of follow-up was defined as either the end of surveillance, 4-years after baseline; date of death; date of internal migration out of the cluster; date of migration out of the study area; or onset date of first severe cholera episode, whichever came first. Person-years of observation (PYO) were calculated from the sum of all follow up periods.

For peer review only

Selection of WASH variables for analyses

We first examined ten household WASH variables ascertained in the demographic censuses and categorized each variable (shared toilet, drinking water source, distance to source of drinking water, drinking water treatment, toilet type, water availability, waste disposal location, hand washing water available, hand washing soap available and shared kitchen) into two categories: "Better" versus "Not Better". The categorization of WASH variables was based on local context-informed judgement and the distribution of WASH variables, but without prior information on cholera incidence rates associated with each variable category.

We randomly divided the "non-intervention arm" population into two subpopulations– 50% of the households into a "training" population and the other 50% into a "validation" population. The training population was used to develop the composite WASH decision tree, and the validation population was subsequently used to cross-validate the decision tree rule. We selected WASH variables associated with risk of severe cholera using a Cox proportional hazard regression model at p-value <0.2 using the training population. We verified that each selected independent variable fulfilled proportionality assumptions before inclusion into the model.

Construction of decision tree to develop composite WASH variable

We developed a composite rule for existing "Better" *versus* "Not Better" household WASH through a machine learning approach to measure the association between WASH status and the incidence of severe cholera. To create a single, binary composite WASH variable predicting the occurrence of severe cholera, we constructed a decision tree with a recursive partitioning approach including variables which were associated with severe cholera.[15]

Given relatively few endpoints compared to the total number of individuals followed, we accounted for the imbalanced case distribution within the construction of the decision tree. The decision tree was designed by assuming a 1:670 loss function for the cost of false positive and false negative classification, and by defining 300 as the minimum number of observations required in each terminal node. The number of cross-validations was 10. We ran the algorithm with the training subpopulation and subsequently pruned the tree by the minimal complexity parameter, corresponding to a minimum error with at least two terminal nodes to make the optimal decision for predicting severe cholera.

With the selected tree, a receiver operating characteristic (ROC) curve was constructed to find the optimal cut-off probability of the composite WASH variable for predicting the occurrence of severe cholera cases. [16,17] This threshold was used to classify the training population with the resulting binary composite WASH variable in relation to the risk of severe cholera: "Better" for lower probability of developing severe cholera, and "Not Better" for higher probability of developing severe cholera.

For peer review only - http://bmjopen.bmj.com/site/about/guidelines.xhtml

To assess the performance of the composite WASH, we applied the same rule to the validation population to confirm that the decision tree exhibited similar sensitivity and specificity in both populations.

Protective association between WASH and severe cholera

Next, we measured the association between "Better" WASH household status and severe cholera in the total population residing in non-intervention clusters. To evaluate the protective association of "Better" WASH, we analyzed the time from start of follow-up to the first severe cholera case using the Cox proportional hazard regression model.

The model was adjusted for potential confounding covariates, including age in years at start of follow-up, sex, and variables reflecting socioeconomic status: monthly expenditure, house ownership, house having one room, and house wall constructed by brick/cement. We identified variables fitted into the model by mixed stepwise selection, using a combination of forward and backward selection with the cutoff p-value of 0.1 for both elimination and retention. Hazard ratios (HR) for severe cholera were estimated by exponentiating the coefficient for the composite WASH variable in models and protection was estimated as [(1- HR) X 100%] with 95% confidence intervals. Estimates were also adjusted for design effect of cluster randomization of the study clusters.

To determine cluster-level "Better" WASH coverage, the person-years of observation of household members living in "Better" WASH households in the cluster were divided by the person-years of the entire population in the same cluster. The association between cluster-level WASH coverage and incidence of severe cholera was assessed after adjustment for potential confounding variables (age, sex, monthly expenditure, house ownership, house having one room and house wall constructed by brick/cement) in proportional hazard models using the same approach.

Statistical analyses were performed using R Studio analytical software for decision tree modeling (rpart package), tree plotting (rpart.plot package), and ROC curve illustration (pROC package). Other statistical analyses were performed using SAS version 9.4. All p values were two-sided.

Results

Training and validation subpopulations

A total of 193,576 individuals in the non-intervention ICVB arm were included in the analysis (Figure 1). Of those, 80,720 individuals were present at baseline and 112,856 individuals were new entrants (107,381 in-migration individuals, 5,475 births). During the four-years of follow-up, a total of 292 severe cholera episodes were observed. The training set was composed of 96,943 individuals, 144 of whom developed severe cholera. The validation set was composed of 96,633 individuals, 148 of whom developed severe cholera. As shown in Table 1,

baseline characteristics of the training and validation subpopulations were broadly comparable in terms of mean age, sex ratio, average monthly expenditure, and household characteristics.

Table 1. Baseline characteristics of the total, training, and validation subpopulations

	Total Population	Training sub-population	Validation sub-population
	n=193576	n=96943	n=96633
Age in years - mean (std)	22.9 (15.4)	22.9 (15.3)	22.9 (15.4)
Gender: Male - n (%)	94008 (48.6)	47365 (48.9)	46643 (48.3)
Monthly Expenditure – mean (std)	10288.6 (5374.1)	10293.1 (4891.7)	10284.1 (5817.7)
Ownership: Own house - n (%)	28677 (14.8)	14549 (15.0)	14128 (14.6)
House: having one room - n (%)	165215 (85.3)	82427 (85.0)	82788 (85.7)
Wall: Brick/Cement - n (%)	139860 (72.3)	69880 (72.1)	69980 (72.4)

[*] std: standard deviation; n: number of individuals

Rule development for composite WASH variable predicting severe cholera case

A bivariate analysis for each variable in the training population was performed to measure associations of individual WASH-related variables with the risk of severe cholera (Table 2). Drinking water source (51% reduction of risk; 95% CI: -20-80), distance to source of drinking water (39% reduction of risk ; 95% CI: 14-57) and drinking water treatment (36% reduction of risk; 95% CI: 11-54) were selected to determine the composite binary WASH variable. The resulting tree, shown in Figure 2, found that treatment of drinking water was the dominant bifurcation; regardless of other WASH variables in the tree, not treating drinking water continually categorized the household as having "Not better" WASH.

Table 2: Bivariate relationship of WASH variables with severe cholera risk in the training subpopulation

WASH Variable (and criteria for Better WASH categorization)	Better WASH				Not Better WASH				Protective association between each variable and severe cholera	
	N	Cases	Person-years	IR (95% CI) Per 100000/PY	N	Cases	Person-years	IR (95% CI) Per 100000/PY	Crude (95%CI)	p-value
**Shared toilet** *No*	4620	9	10253	88 (46, 169)	92323	135	134076	101 (85, 119)	21 (-55, 60)	0.49
**Toilet type** *Sanitary latrine with or without flush*	80974	122	118747	103 (86, 123)	15969	22	25582	86 (57, 131)	-24 (-95, 21)	0.36
**Drinking Water Source:** *Own tap/well/pump/bottled or vendor water*	4187	5	8993	56 (23, 134)	92756	139	135337	103 (87, 121)	51 (-20, 80)	0.12

For peer review only

**Water availability:**<br>*Tap/tube well/well water is available all the time*	67831	95	93293	102 (83, 125)	29112	49	51036	96 (73, 127)	-15 (-64, 19)	0.43
**Distance to source of drinking water**<br>*Using median as cut off*	43806	47	64461	73 (55, 97)	53137	97	79869	121 (100, 148)	39 (14, 57)	<0.01
**Drinking Water Treatment:**<br>*Filtered/Boiled/chemical treated*	54275	60	77380	78 (60, 100)	42668	84	66950	125 (101, 155)	36 (11, 54)	<0.01
**Waste disposal location**: *Fixed*	80198	109	116056	94 (78, 113)	16745	35	28273	124 (89, 172)	20 (-17, 46)	0.25
**Hand Washing Water Available***<br>*Yes*	93758	138	138159	100 (85, 118)	3185	6	6170	97 (44, 216)	-11 (-152, 51)	0.8
**Hand Washing Soap Available***<br>*Yes*	91514	133	135316	98 (83, 117)	5429	11	9014	122 (68, 220)	17 (-54, 55)	0.56
**Shared kitchen**<br>*No*	86713	118	120182	98 (82, 118)	10230	26	24147	108 (73, 158)	-5 (-62, 32)	0.83

Performance of the composite WASH variable predicting severe cholera

In the training set, we found that an optimal cut-off value of 0.0012 for the composite WASH variable was found to maximize the Youden index using the ROC curve, with an area under the curve (AUC) of 59% (95% CI: 55-63) (Figure 3). Under this threshold, the rule predicted 123 true positives of 144 severe cholera episodes, for a sensitivity of 85% (95% CI: 85-86), and 28,709 true negatives among 96,799 persons without severe cholera, yielding a specificity of 30% (95% CI: 22-37). The composite WASH variable performed similarly when applied to the validation set, with a sensitivity of 82% (95% CI: 82-82) and specificity of 30% (95% CI: 22-37).

Prediction of severe cholera incidence by household WASH status in the total population

We applied the WASH prediction rule to the total population residing in the non-intervention clusters to predict severe cholera episode risk. 29.7% of households in the non-intervention arm were classified as having "Better" WASH and the remaining 70.3% were classified as having "Not Better" WASH.

The incidence of severe cholera in all age groups living in the "Better" WASH households was 57 per 100,000 person-years of observation (PYO), compared to 120 per 100,000 PYO in "Not Better" WASH households, a 47% (95% CI: 29-61, $p<0.001$) reduced risk of severe cholera, after adjusting for covariates. The protective association between living in a "Better" WASH households and severe cholera risk was consistent in all age groups (Table 3). For individuals under the age of 15, living in a "Better" WASH households was associated with

For peer review only

having 64% (95% CI: 36-80; *p<0.001*) reduced risk of severe cholera. The protective association of "Better" WASH household status was comparatively lower in the 15+ age-group, where "Better" WASH was associated with a 43% (95% CI: 16-61; *p=0.004*) reduction in severe cholera risk.

Table 3. Protective association between "Better" WASH and severe cholera in the total study population

	"Better" WASH				"Not Better" WASH				Protective association between "Better" WASH and severe cholera			
	n	cases	Person-years	IR per 100000/PYs (95% CI)	N	cases	Person-years	IR per 100000/PYs (95% CI)	Crude (95%CI)	p-value	Adjusted* (95%CI)	p-value
**All**	57396	48	84817	57 (43, 75)	136180	244	203701	120 (106, 136)	52 (34, 65)	<0.001	47 (29, 61)	<0.001
<5 years	7552	5	10518	48 (20, 114)	17149	42	24366	172 (127, 233)	72 (25, 89)	0.012	69 (15, 88)	0.022
5-14 years	8861	4	14618	27 (10, 73)	25318	32	42389	75 (53, 107)	63 (6, 85)	0.037	63 (6, 85)	0.037
15+ years	40983	39	59680	65 (48, 89)	93713	170	136945	124 (107, 144)	46 (20, 64)	0.002	43 (16, 61)	0.004

** Adjusted for design effect and selected covariates by stepwise selection using cutoff 0.1 for both of elimination and addition; For entire population, selected covariates were age, gender, home ownership and house having one room; For <5 years, selected covariate was home ownership; For 5-14 years, no covariate was selected; For 15+ years, selected covariates were age, gender and house having one room;*

There was a slight, non-significant negative association between rising proportion of "Better" WASH households in a cluster and incidence rate of severe cholera (Figure 4). In our model, for every ten percent increase in proportion of "Better" WASH households in a cluster, individuals living in that cluster experienced 4% lower risk of severe cholera, though this relationship was not significant (HR=0.996 (95% CI: 0.988-1.005; *p-value: 0.40*).

Discussion

Using a machine learning approach, we developed a composite "Better" WASH variable to predict severe cholera risk using data from a prospective cohort study in a Dhaka slum. The "Better" WASH decision tree found that water quality and access were the most significant factors associated with severe cholera risk.

This finding is consistent with previous literature and findings from Wolfe and colleagues' systematic review of case-control studies that found strong associations between

For peer review only - http://bmjopen.bmj.com/site/about/guidelines.xhtml

household water quality and lowered incidence of cholera.[18] In this review of mostly epidemic cholera contexts, the authors found that eight WASH-related risk factors, including unimproved water source, untreated water, unsafe water storage and transport, were consistently associated with higher odds of cholera. As such, the importance of water practices in our determination of "Better" WASH is in line with the prevailing understanding of cholera risk.

In our analysis, we found that individuals living in "Better" WASH households had 47% (95% CI: 29-61) reduced risk of severe cholera compared to individuals living in "Not Better" WASH households, after adjusting for age, gender, and socioeconomic factors. This statistically significant protective association was demonstrated in all age groups examined, though those under-15 exhibited a greater degree of protection compared to those over-15. This difference by age group may stem from the differences in WASH behaviors and exposure to cholera—wherein individuals 15 and older are more likely to have many exposures outside of the household at school, work, or play that could reduce the protective effects of living in a "Better" WASH home.

There are several strengths to our analysis that lend credibility to the findings. The analysis was evaluated in the context of a prospective CRT in a well-defined population with comprehensive follow-up for cholera detection. We followed this population for four-years, making it one of the longest evaluations of the relationship between household WASH and severe cholera incidence, and thereby shedding light on the long-term durability of WASH adaptations. Further, during the development of the composite WASH rule we reduced bias by categorizing WASH variables without any prior knowledge of cholera incidence rate associated with each component category. We also rigorously validated the WASH prediction rule by using a validation sub-population to ensure that the rule was not over-fitted to the training sub-population.

Our analysis also has limitations. Firstly, household WASH variables were only evaluated once and applied to the whole study period. For the population present at baseline, household WASH status was evaluated once at the beginning of the study and applied to the entire four-year study period. As household WASH variables are more likely to improve rather than regress over time as household members age and income increases, this misclassification bias should have led to more conservative estimates of protective associations. Furthermore, it must be noted that the household WASH variables included in this study were collected in the context of an OCV trial through a brief questionnaire designed to assess confounding variables in the evaluation of vaccine effectiveness. This approach may have led to loss of information, making the WASH-cholera relationship conservative, and may also have identified variables that are "proxies" for actual water-related factors that directly mediate a reduced risk of cholera. Consequently, the WASH variables included in the composite rule may best be interpreted as

For peer review only - http://bmjopen.bmj.com/site/about/guidelines.xhtml

markers of these direct mediators. Nonetheless, because these variables predicted severe cholera risk independently of non-WASH socioeconomic factors, the analysis underscores the importance of water-related WASH adaptations in determining the risk of severe cholera in this setting.

Our findings indicate that there are existing culturally acceptable WASH improvements that can be impactful in controlling cholera in a dense slum population considered to have hyperendemic cholera. Past experiences in WASH intervention programs have shown that achieving sustainable and effective WASH interventions can be challenging. For example, an evaluation of a household water treatment and handwashing campaign in rural Guatemala found that three years after the intervention there were no differences in handwashing behavior, WASH conditions, or prevalence of childhood diarrhea in the community[19]. Other examples of poor long-term uptake and acceptability of WASH interventions are found in programs implemented in India, Zambia, and Kenya—all highlighting the difficulty of sustained behavior adoption.[20–22] In fact in the ICVB trial from which this analysis originates, the WASH intervention was found to *not* improve effectiveness of OCV when combined, likely because of poor uptake in the community. In light of this, understanding *existing* "Better" WASH households and behaviors in endemic slum communities can provide valuable lessons on feasible and sustainable interventions.

Recent studies have also found that Mirpur, Bangladesh, the site for this study, has very high rates of microbiological proliferation and contamination in the municipal water supply,[23] making achievable WASH improvements paramount to improving community health. Our analysis finds that there are existing household water adaptations in Mirpur that are associated with significantly lowered severe cholera risk, despite contaminated water at the municipal level. The fact that the WASH adaptations practiced by 30% of the population were significantly predictive of lower cholera risk even within slum conditions speaks to the potential for existing knowledge to inform cholera control strategies.

Cholera transmission in urban slums may only intensify with the pressures of rapid urbanization combined with climate change effects on temperature and precipitation. Endemic cholera in urban areas is a reality in Bangladesh that faces different challenges from previously studied epidemic and rural cholera transmission. Closely examining how some urban households have already made WASH adaptations to reduce cholera risk and leveraging existing practices in the slums will help design effective cholera control programs that are sustainable and achievable.

For peer review only - http://bmjopen.bmj.com/site/about/guidelines.xhtml

Data Availability Statement

Data will be made available in a public, open access repository.

Ethics Statement

This study is a re-analysis of data from a cluster randomized trial entitled 'Introduction of cholera vaccine in Bangladesh (ICVB)' conducted by the icddr,b in 2011. The ICVB trial received Institutional Review Board approval (icddr,b protocol number PR#17124) and Bangladesh National Research Ethics Committee approval. Participants gave informed consent to participate in the study before taking part.

Author Contributions

JDC conceptualized the study. SK and FC prepared the original draft. Data analysis was carried out by JP, FA, and DRK. Additionally JDC, FQ, FM, JHK, and AIK supervised the study. All authors participated in the writing of the manuscript and interpreting the data. All authors reviewed and approved the final manuscript.

Competing Interests

There are no competing interests from any of the authors.

Funding

This work was supported, in whole or in part, by the Bill & Melinda Gates Foundation [INV 025386]. Under the grant conditions of the Foundation, a Creative Commons Attribution 4.0 Generic License has already been assigned to the Author Accepted Manuscript version that might arise from this submission.

Acknowledgement

Icddr,b is grateful to the Governments of Bangladesh, Canada, Sweden and the UK for providing unrestricted support. Thanks to Dr. Nigel McMillan (Griffith University) for his scientific and technical support in the manuscript. The funders had no role in study design, data collection and analysis, decision to publish, or preparation of the manuscript.

Figure Legends

Figure 1. CONSORT- Dynamic population during 4-year follow up period

Figure 2. Rule predicting severe cholera episode risk in the training subpopulation

Figure 3. ROC curve for the performance of the decision rule in the training subpopulation

Figure 4. Relationship between cluster-level "Better" WASH coverage and risk of severe cholera

For peer review only

For peer review only

References

1. Ritchie, H. & Roser, M. Urbanization. *Our World Data* (2018).

2. Dhaka: fastest growing megacity in the world. *The World from PRX* https://www.pri.org/stories/2010-09-08/dhaka-fastest-growing-megacity-world.

3. Ross, A. G. P., Zaman, K. & Clemens, J. D. Health Concerns in Urban Slums: A Glimpse of Things to Come? *JAMA* **321**, 1973–1974 (2019).

4. Islam, M. T., Clemens, J. D. & Qadri, F. Cholera Control and Prevention in Bangladesh: An Evaluation of the Situation and Solutions. *J. Infect. Dis.* **218**, S171–S172 (2018).

For peer review only - http://bmjopen.bmj.com/site/about/guidelines.xhtml

5. Longini, I. M., Jr. *et al.* Epidemic and Endemic Cholera Trends over a 33-Year Period in Bangladesh. *J. Infect. Dis.* **186**, 246–251 (2002).

6. Asadgol, Z. *et al.* How climate change can affect cholera incidence and prevalence? A systematic review. *Environ. Sci. Pollut. Res.* **27**, 34906–34926 (2020).

7. Salerno, F., Gaetano, V. & Gianni, T. Urbanization and climate change impacts on surface water quality: Enhancing the resilience by reducing impervious surfaces. *Water Res.* **144**, 491–502 (2018).

8. Kumar, M., Deka, J. P. & Kumari, O. Development of Water Resilience Strategies in the context of climate change, and rapid urbanization: A discussion on vulnerability mitigation. *Groundw. Sustain. Dev.* **10**, 100308 (2020).

9. Taylor, D. L., Kahawita, T. M., Cairncross, S. & Ensink, J. H. J. The Impact of Water, Sanitation and Hygiene Interventions to Control Cholera: A Systematic Review. *PLOS ONE* **10**, e0135676 (2015).

10. George, C. M. *et al.* Randomized Controlled Trial of Hospital-Based Hygiene and Water Treatment Intervention (CHoBI7) to Reduce Cholera. *Emerg. Infect. Dis.* **22**, 233–241 (2016).

11. Luby, S. P., Davis, J., Brown, R. R., Gorelick, S. M. & Wong, T. H. F. Broad approaches to cholera control in Asia: Water, sanitation and handwashing. *Vaccine* **38 Suppl 1**, A110–A117 (2020).

12. Childs, L. *et al.* Evaluation of Knowledge and Practices Regarding Cholera, Water Treatment, Hygiene, and Sanitation Before and After an Oral Cholera Vaccination Campaign-Haiti, 2013-2014. *Am. J. Trop. Med. Hyg.* **95**, 1305–1313 (2016).

For peer review only

13. Qadri, F. *et al.* Feasibility and effectiveness of oral cholera vaccine in an urban endemic setting in Bangladesh: a cluster randomised open-label trial. *The Lancet* **386**, 1362–1371 (2015).

14. Khan, I. A. *et al.* Organization and implementation of an oral cholera vaccination campaign in an endemic urban setting in Dhaka, Bangladesh. *Glob. Health Action* **12**, (2019).

15. Strobl, C., Malley, J. & Tutz, G. An Introduction to Recursive Partitioning: Rationale, Application and Characteristics of Classification and Regression Trees, Bagging and Random Forests. *Psychol. Methods* **14**, 323–348 (2009).

16. Hand, D. J. Measuring classifier performance: a coherent alternative to the area under the ROC curve. *Mach. Learn.* **77**, 103–123 (2009).

17. Faraggi, D. & Reiser, B. Estimation of the area under the ROC curve. *Stat. Med.* **21**, 3093–3106 (2002).

18. Wolfe, M., Kaur, M., Yates, T., Woodin, M. & Lantagne, D. A Systematic Review and Meta-Analysis of the Association between Water, Sanitation, and Hygiene Exposures and Cholera in Case–Control Studies. *Am. J. Trop. Med. Hyg.* **99**, 534–545 (2018).

19. Arnold, B., Arana, B., Mäusezahl, D., Hubbard, A. & Colford, J. M., Jr. Evaluation of a pre-existing, 3-year household water treatment and handwashing intervention in rural Guatemala. *Int. J. Epidemiol.* **38**, 1651–1661 (2009).

For peer review only

20. Makutsa, P. *et al.* Challenges in Implementing a Point-of-Use Water Quality Intervention in Rural Kenya. *Am. J. Public Health* **91**, 1571–1573 (2001).

21. Firth, J. *et al.* Point-of-Use Interventions to Decrease Contamination of Drinking Water: A Randomized, Controlled Pilot Study on Efficacy, Effectiveness, and Acceptability of Closed Containers, Moringa oleifera, and In-home Chlorination in Rural South India. *Am. J. Trop. Med. Hyg.* **82**, 759–765 (2010).

22. Peletz, R. *et al.* Follow-Up Study to Assess the Use and Performance of Household Filters in Zambia. *Am. J. Trop. Med. Hyg.* **89**, 1190–1194 (2013).

23. Acharjee, M., Rahman, F., Jahan, F. & Noor, R. Bacterial Proliferation in Municipal Water Supplied in Mirpur Locality of Dhaka City, Bangladesh: Microbiological Study of Municipal Water. *CLEAN - Soil Air Water* **42**, 434–441 (2014).

For peer review only

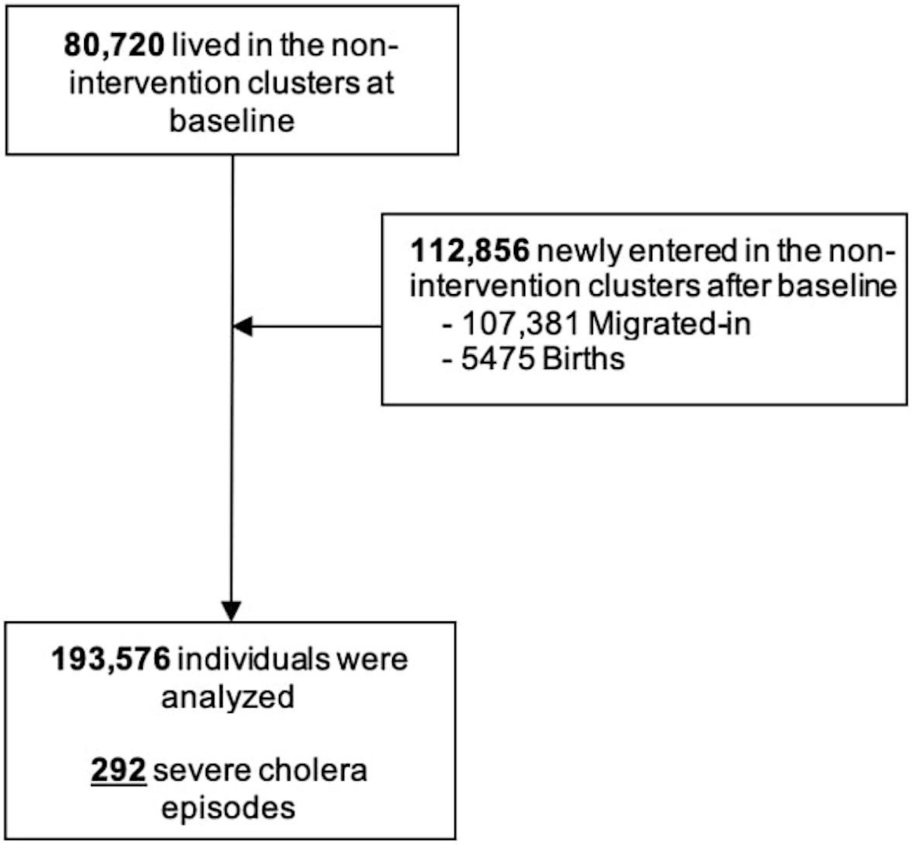

Figure 1: CONSORT- Dynamic population during 4-year follow up period

109x102mm (300 x 300 DPI)

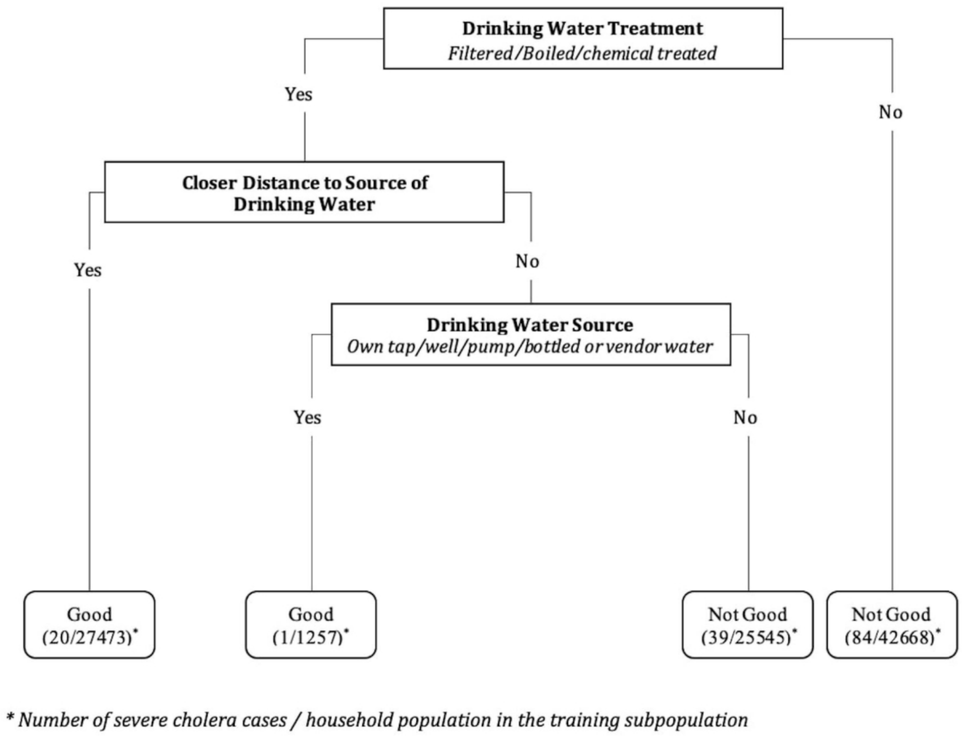

Figure 2. Rule predicting severe cholera episode risk in the training subpopulation

150x115mm (300 x 300 DPI)

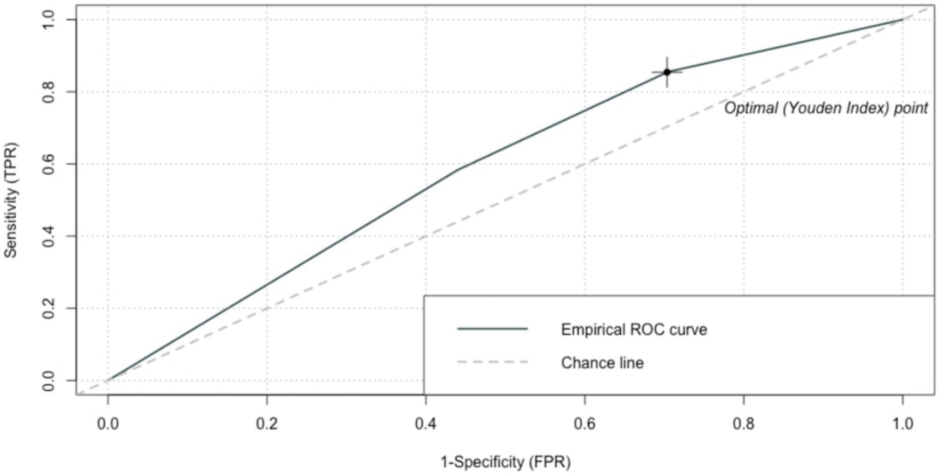

AUC = 59% (95% CI, 55-63); optimal cut-off probability using the Youden index = 0.0012

Figure 3. ROC curve for the performance of the decision rule in the training subpopulation

180x100mm (300 x 300 DPI)

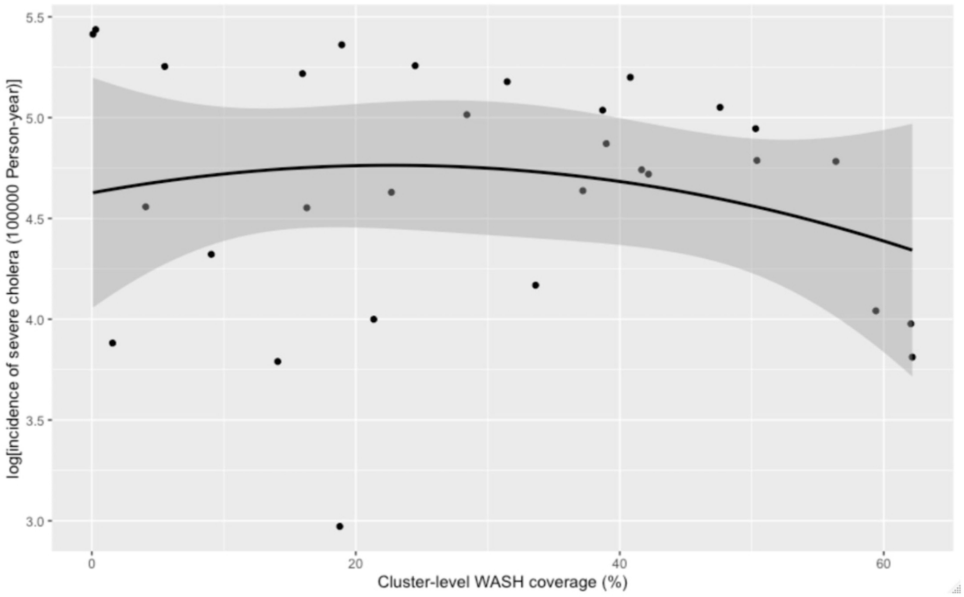

Figure 4. Relationship between cluster-level “Better” WASH coverage and risk of severe cholera

189x119mm (300 x 300 DPI)

STROBE Statement—Checklist of items that should be included in reports of ***cohort studies***

	Item No	Recommendation	Page No
**Title and abstract**	1	(*a*) Indicate the study's design with a commonly used term in the title or the abstract	1
		(*b*) Provide in the abstract an informative and balanced summary of what was done and what was found	2
**Introduction**			
Background/rationale	2	Explain the scientific background and rationale for the investigation being reported	4
Objectives	3	State specific objectives, including any prespecified hypotheses	4
**Methods**			
Study design	4	Present key elements of study design early in the paper	1-2
Setting	5	Describe the setting, locations, and relevant dates, including periods of recruitment, exposure, follow-up, and data collection	2, 4-5
Participants	6	(*a*) Give the eligibility criteria, and the sources and methods of selection of participants. Describe methods of follow-up	5-6
		(*b*) For matched studies, give matching criteria and number of exposed and unexposed	N/A
Variables	7	Clearly define all outcomes, exposures, predictors, potential confounders, and effect modifiers. Give diagnostic criteria, if applicable	5-6
Data sources/ measurement	8*	For each variable of interest, give sources of data and details of methods of assessment (measurement). Describe comparability of assessment methods if there is more than one group	5-7
Bias	9	Describe any efforts to address potential sources of bias	6-7
Study size	10	Explain how the study size was arrived at	N/A
Quantitative variables	11	Explain how quantitative variables were handled in the analyses. If applicable, describe which groupings were chosen and why	6-7
Statistical methods	12	(*a*) Describe all statistical methods, including those used to control for confounding	6-8
		(*b*) Describe any methods used to examine subgroups and interactions	6-8
		(*c*) Explain how missing data were addressed	N/A
		(*d*) If applicable, explain how loss to follow-up was addressed	6
		(*e*) Describe any sensitivity analyses	N/A
**Results**			
Participants	13*	(a) Report numbers of individuals at each stage of study—eg numbers potentially eligible, examined for eligibility, confirmed eligible, included in the study, completing follow-up, and analysed	8
		(b) Give reasons for non-participation at each stage	N/A
		(c) Consider use of a flow diagram	12
Descriptive data	14*	(a) Give characteristics of study participants (eg demographic, clinical, social) and information on exposures and potential confounders	13
		(b) Indicate number of participants with missing data for each variable of interest	N/A
		(c) Summarise follow-up time (eg, average and total amount)	8
Outcome data	15*	Report numbers of outcome events or summary measures over time	8

For peer review only

Main results	16	(*a*) Give unadjusted estimates and, if applicable, confounder-adjusted estimates and their precision (eg, 95% confidence interval). Make clear which confounders were adjusted for and why they were included	9
		(*b*) Report category boundaries when continuous variables were categorized	N/A
		(*c*) If relevant, consider translating estimates of relative risk into absolute risk for a meaningful time period	N/A
Other analyses	17	Report other analyses done—eg analyses of subgroups and interactions, and sensitivity analyses	11
**Discussion**			
Key results	18	Summarise key results with reference to study objectives	10
Limitations	19	Discuss limitations of the study, taking into account sources of potential bias or imprecision. Discuss both direction and magnitude of any potential bias	12
Interpretation	20	Give a cautious overall interpretation of results considering objectives, limitations, multiplicity of analyses, results from similar studies, and other relevant evidence	11-13
Generalisability	21	Discuss the generalisability (external validity) of the study results	12-13
**Other information**			
Funding	22	Give the source of funding and the role of the funders for the present study and, if applicable, for the original study on which the present article is based	13

*Give information separately for exposed and unexposed groups.

Note: An Explanation and Elaboration article discusses each checklist item and gives methodological background and published examples of transparent reporting. The STROBE checklist is best used in conjunction with this article (freely available on the Web sites of PLoS Medicine at http://www.plosmedicine.org/, Annals of Internal Medicine at http://www.annals.org/, and Epidemiology at http://www.epidem.com/). Information on the STROBE Initiative is available at http://www.strobe-statement.org.

For peer review only

For peer review only - http://bmjopen.bmj.com/site/about/guidelines.xhtml

BMJ Open

Are better existing WASH practices in urban slums associated with a lower long-term risk of severe cholera? A prospective cohort study with four years of follow-up in Mirpur, Bangladesh

Journal:	*BMJ Open*
Manuscript ID	bmjopen-2022-060858.R1
Article Type:	Original research
Date Submitted by the Author:	30-May-2022
Complete List of Authors:	Kang, Sophie; International Vaccine Institute, Epidemiology, Public Health, and Impact Unit<br>Chowdhury, Fahima; International Centre for Diarrhoeal Disease Research Bangladesh; Griffith University Menzies Health Institute Queensland<br>Park, Juyeon; International Vaccine Institute, Epidemiology, Public Health, and Impact Unit; University of Cambridge, Department of Medicine<br>Ahmed, Tasnuva; International Centre for Diarrhoeal Disease Research Bangladesh<br>Tadesse, Birkneh Tilahun; International Vaccine Institute, Epidemiology, Public Health, and Impact Unit<br>Islam, Md. Taufiqul; International Centre for Diarrhoeal Disease Research Bangladesh<br>Kim, Deok Ryun; International Vaccine Institute, Epidemiology, Public Health, and Impact Unit<br>Im, Justin; International Vaccine Institute<br>Aziz, Asma; International Vaccine Institute, Epidemiology, Public Health, and Impact Unit<br>Hoque, Masuma; International Centre for Diarrhoeal Disease Research Bangladesh<br>Pak, GiDeok; International Vaccine Institute<br>Khanam, Farhana; International Centre for Diarrhoeal Disease Research Bangladesh<br>Ahmmed, Faisal; International Centre for Diarrhoeal Disease Research Bangladesh<br>Liu , Xinxue ; University of Oxford Oxford Vaccine Group<br>Zaman, K; International Centre for Diarrhoeal Disease Research Bangladesh<br>Khan, Ashraful Islam; International Centre for Diarrhoeal Disease Research<br>Kim, Jerome ; International Vaccine Institute<br>Marks, Florian ; International Vaccine Institute; University of Cambridge, Department of Medicine<br>Qadri, Firdausi ; International Centre for Diarrhoeal Disease Research Bangladesh<br>Clemens, John; International Vaccine Institute, Epidemiology, Public Health, and Impact Unit; International Centre for Diarrhoeal Disease

	Research Bangladesh
**Primary Subject Heading**:	Global health
Secondary Subject Heading:	Epidemiology, Infectious diseases, Public health
Keywords:	EPIDEMIOLOGY, Epidemiology < INFECTIOUS DISEASES, INFECTIOUS DISEASES, PUBLIC HEALTH

SCHOLARONE™
Manuscripts

I, the Submitting Author has the right to grant and does grant on behalf of all authors of the Work (as defined in the below author licence), an exclusive licence and/or a non-exclusive licence for contributions from authors who are: i) UK Crown employees; ii) where BMJ has agreed a CC-BY licence shall apply, and/or iii) in accordance with the terms applicable for US Federal Government officers or employees acting as part of their official duties; on a worldwide, perpetual, irrevocable, royalty-free basis to BMJ Publishing Group Ltd ("BMJ") its licensees and where the relevant Journal is co-owned by BMJ to the co-owners of the Journal, to publish the Work in this journal and any other BMJ products and to exploit all rights, as set out in our licence.

The Submitting Author accepts and understands that any supply made under these terms is made by BMJ to the Submitting Author unless you are acting as an employee on behalf of your employer or a postgraduate student of an affiliated institution which is paying any applicable article publishing charge ("APC") for Open Access articles. Where the Submitting Author wishes to make the Work available on an Open Access basis (and intends to pay the relevant APC), the terms of reuse of such Open Access shall be governed by a Creative Commons licence – details of these licences and which Creative Commons licence will apply to this Work are set out in our licence referred to above.

Other than as permitted in any relevant BMJ Author's Self Archiving Policies, I confirm this Work has not been accepted for publication elsewhere, is not being considered for publication elsewhere and does not duplicate material already published. I confirm all authors consent to publication of this Work and authorise the granting of this licence.

For peer review only

Are better existing WASH practices in urban slums associated with a lower long-term risk of severe cholera? A prospective cohort study with four years of follow-up in Mirpur, Bangladesh

Sophie Kang[1*], Fahima Chowdhury[2,7*], Juyeon Park[1,4], Tasnuva Ahmed[2], Birkneh Tilahun Tadesse[1], Md. Taufiqul Islam[2], Deok Ryun Kim[1], Justin Im[1], Asma Binte Aziz[1], Masuma Hoque[2], Gideok Pak[1], Farhana Khanam[2], Faisal Ahmmed[2], Xinxue Liu[3], Khalequ Zaman[2], Ashraful Islam Khan[2], Jerome H. Kim[1], Florian Marks[1,4,5], Firdausi Qadri[2], John D. Clemens[1,2,6]

1 International Vaccine Institute, Seoul, Republic of Korea

2 International Centre for Diarrheal Disease Research, Bangladesh, Dhaka, Bangladesh

3 Oxford Vaccine Group, Department of Pediatrics, University of Oxford, Oxford OX3 9DU, UK

4 Department of Medicine, University of Cambridge, Cambridge, United Kingdom

5 University of Antananarivo, Antananarivo, Madagascar

6 UCLA Fielding School of Public Health, Los Angeles, CA 90095-1772, USA

7 Menzies Health Institute Queensland, Griffith University, Gold Coast, Australia

* Contributed equally

Correspondence to Sophie Kang, International Vaccine Institute; Sophie.kang@ivi.int

For peer review only

For peer review only

Abstract

Objective

To investigate the association between existing household WASH practices and severe cholera risk in a dense urban slum where cholera is highly endemic.

Design, Setting, and Participants

We assembled a large prospective cohort within a cluster randomized trial evaluating the effectiveness of oral cholera vaccine. Our dynamic cohort population (n=193,576) comprised individuals living in the "non-intervention" clusters of the trial, and were followed over four years. This study was conducted in a dense urban slum community of Dhaka, Bangladesh and cholera surveillance was undertaken in twelve hospitals serving the study area.

Primary outcome measure

First severe cholera episode detected during follow-up period.

Methods

We applied a machine learning algorithm on a training subpopulation (n=96,943) to develop a binary ("Better", "Not Better") composite WASH variable predictive of severe cholera. The WASH rule was evaluated for performance in a separate validation subpopulation (n=96,633). Afterwards, we used Cox-regression models to evaluate the association between "Better" WASH households and severe cholera risk over 4 years in the entire study population.

Results

The "Better" WASH rule found that water quality and access were the most significant factors associated with severe cholera risk. Members of "Better" WASH households, constituting one-third of the population, had a 47% reduced risk of severe cholera (95% CI: 29-69; *p*-value <0.001), after adjusting for covariates. The protective association between living in a "Better" WASH household and severe cholera persisted in all age groups.

Conclusions

Salutary existing household WASH practices were associated with a significantly reduced long-term risk of severe cholera in an urban slum of Dhaka. These findings suggest that WASH adaptations already practiced in the community may be important for developing and implementing effective and sustainable cholera control programs in similar settings.

Strengths and limitations of this study

- We studied a cohort prospectively followed for four years, making this analysis one of the longest uninterrupted evaluations of the relationship between household WASH and endemic cholera.
- The multivariable WASH prediction rule was rigorously validated using a separate sub-population and thus avoided overfitting to the training set.
- We focused on the existing variability of household WASH within a dense slum community to demonstrate that there are salutary practices that may reduce the long-term risk of severe cholera.
- Household WASH factors were only evaluated once, either at baseline or when participants entered the study area, and this status applied to the entire follow-up period .
- The household WASH variables included in this study were collected in the context of an OCV trial and not optimized for describing WASH factors independently.

For peer review only

Introduction

The developing world has seen a rapid expansion of urban areas due, in part, to influxes into squalid urban slums. With an estimated 55% of the world's population living in urban areas, 1-in-3 of those urban dwellers live in slum households [1]. Slum households are defined as those which lack one or more of the following conditions: access to improved water, access to improved sanitation, sufficient living area, and durability of housing.

In Bangladesh urban dwellers currently account for 38% of the population and are expected to exceed 50% by 2030; furthermore, 47% of the urban population lives in slums, where residents are at increased risk for waterborne diseases, including cholera [2,3]. Cholera is a major cause of morbidity and mortality in Bangladesh [4]. Previously thought to be a rural disease in Bangladesh, cholera is now becoming a disease of cities and slums where living conditions create different challenges for cholera control from those encountered in rural settings [5]. Lessons learned from rural areas, and particularly in epidemic situations, may not be applicable to the changing pattern of cholera endemicity in urban areas. Specific studies on how endemic cholera can be controlled in these urban slums are needed.

Exacerbating the urban cholera situation further is the role of climate change. Rising temperatures and increased precipitation associated with climate change are significant

For peer review only

predictors of cholera incidence, with strong evidence in studies of Bangladesh where warmer, wetter conditions are associated with major cholera outbreaks [6]. Climate change effects in urban areas have added negative implications for water quality, and studies have found that increased rainfall intensity combined with impervious urban surfaces are significant predictors of sewer overflows that greatly impact water quality [7,8].

Improvements of water quality, sanitation, and hygiene (WASH) and oral cholera vaccine (OCV) are the major tools for the prevention of endemic cholera, including in urban slums. However, while WASH interventions are frequently employed to control cholera, evidence regarding their effectiveness is inconsistent and successful implementation may be stymied by limited cultural acceptability, low uptake, and poor community acceptance [9, 10, 11, 12]. Our cluster, randomized trial (CRT) of OCV and WASH in an urban Dhaka slum, which failed to demonstrate that WASH added to protection against severe cholera by OCV, is illustrative [13].

In this re-analysis of the CRT, we followed households in the 'non-intervention' arm to investigate how existing WASH practices and adaptations in the slums may be associated with lower severe cholera risk. We assessed long-term severe cholera risk over four years and sought to identify the salutary practices that might inform the development of future effective, acceptable, and sustainable WASH interventions in cholera-endemic urban populations.

Methods

Trial and Population

In 2011, the International Centre for Diarrheal Disease Research, Bangladesh (icddr,b) conducted a cluster randomized control trial entitled 'Introduction of cholera vaccine in Bangladesh (ICVB)' [13] in six selected wards of Mirpur, Dhaka to evaluate the feasibility and effectiveness of oral cholera vaccine (OCV), both deployed alone and in conjunction with WASH interventions. Vaccination with Shanchol, a two-dose OCV was carried out between 17 February and 6 April, 2011.

Households were grouped into 90 geographic clusters with an average population of 2988 households per cluster (ranging from 2288 to 4299 households per cluster). Clusters were

For peer review only - http://bmjopen.bmj.com/site/about/guidelines.xhtml

randomly assigned (1:1:1) to one of three arms: a two-dose regimen of OCV alone, OCV with a WASH intervention, or no intervention (control) [13]. The two doses of OCV were administered at a 14-day interval. The WASH intervention consisted of a behavioral change intervention which focused on use of a household handwashing station and a chlorine dispenser for the treatment of household drinking water[13]. Healthy, non-pregnant individuals aged one year or older were eligible for vaccination in this cluster randomized trial (CRT), and each cluster was separated from the adjoining cluster by at least a 30-meter buffer area to minimize diffusion of the WASH messages between clusters.

For peer review only

Demographic Surveillance

The study population was characterized with a baseline demographic census and recurring census updates to surveille births, deaths, and migrations in the community. The baseline demographic census was conducted before the start of the ICVB vaccination campaign and updated bi-annually. Verbal consent for participation in the surveillance was obtained and documented in a questionnaire at the time of the baseline census and at each bi-annual update. A "household" was defined as persons sharing the same cooking pot.

In addition to the basic demographic information, household-level socioeconomic status, WASH data, and geographical locations of each household were collected during the baseline census. For households that were not present at the baseline census, WASH characteristics were assessed at the first bi-annual census update they were captured in. Household WASH characteristics were not re-assessed for new births in a household already characterized during the study period. All individuals living in the study area were provided with a study identification card containing the unique participant identification (PID) number that was recorded in computerized study databases.

Disease Surveillance

Surveillance for cholera was conducted between April 2011 to November 2015 at two icddr,b hospitals and ten hospitals serving the study area shown in Figure 1. Study surveillance

staff were present at each health facility throughout the day to facilitate reporting of diarrheal cases from the study area. Patients from the study area were identified in the treatment centers with their study identification card or by searching their identities in on-site computerized census database. Clinical examination was carried out by physicians, and designated study staff completed data forms and obtained fecal specimens after obtaining written informed consent.

A diarrheal visit was defined as having 3 or more loose stools or, 1-2 or an indeterminate number of loose stools with evidence of dehydration, in the 24 hours before presentation[13]. If the date of discharge from an earlier diarrheal visit and the date of symptom onset for the subsequent diarrheal visit were within 7 days of one another, then both visits were considered part of the same diarrheal episode. The onset of a diarrheal episode was defined as starting on the day the patient first reported loose or liquid stools.

Fecal samples were examined for *V. cholerae* O1 or O139 serogroups, biotype, and Inaba and Ogawa serotypes using conventional methods[13]. A cholera episode was defined as a diarrheal episode in which a fecal specimen yielded *V. cholerae* O1 or O139, with no passage of bloody stools during the episode. Severely dehydrating cholera was defined by the presence of at least two of the following symptoms or signs of severe dehydration: sunken eyes, dry tongue, thirst, irritable condition, less active than usual along with inability to drink, skin pinch goes back slowly, and low volume of radial pulse[13]. A severe cholera episode was one in which the patient exhibited severe dehydration during any visit of the episode. The primary outcome in this analysis was the first severe cholera episode detected during follow-up.

<u>Patient and Public Involvement</u>

This analysis utilizes data that originates from the ICVB cluster randomized trial conducted by the icddr,b in 2011. Given that ten years have passed since the original study, the participants were not directly involved in developing or informing the design of the analysis described in this paper. That said, the original ICVB trial involved strong social mobilization and community engagement to improve the conduct of the study.

For peer review only - http://bmjopen.bmj.com/site/about/guidelines.xhtml

The research questions addressed in the ICVB cluster randomized trial were developed due to the pressing need to understand the impact of oral cholera vaccines in urban Bangladesh. Advocacy meetings with local government representatives, pediatric associations, and NGOs were held in order to inform the design and conduct of the ICVB trial.

Analytical Approach

Population Under Follow-up

We considered a dynamic population for this analysis, which included the population present in the non-intervention arm at baseline and new entrants into the non-intervention study area during the study period. For those present at baseline, the start of follow-up was defined as the median date of first Shanchol dose in the nearest intervention cluster. For new residents, the start of follow up was defined as either the date of birth or the date of migration into the study area. The end of follow-up was defined as either the end of surveillance, 4-years after baseline; date of death; date of migration out of the cluster; or onset date of first severe cholera episode, whichever came first. Person-years of observation (PYO) were calculated from the sum of follow up periods for individuals under analysis.

Selection of WASH variables for analyses

We first examined the ten household WASH variables ascertained in the demographic censuses and categorized each variable (shared toilet, drinking water source, distance to source of drinking water, drinking water treatment, toilet type, water availability, waste disposal location, hand washing water available, hand washing soap available, and shared kitchen into two categories: “Better” versus “Not Better”. The categorization of WASH variables was based on local context-informed judgement and the distribution of the study population into categories of the WASH variables, but without prior information on cholera incidence rates associated with each variable category.

We randomly divided the population of clusters of the “non-intervention arm” of the trial into two subpopulations– 50% of the households into a “training” population and the other 50%

For peer review only - http://bmjopen.bmj.com/site/about/guidelines.xhtml

into a "validation" population. The training population was used to develop a recursive portioning tree to define the composite WASH variable, and the validation population was subsequently used to cross-validate the decision tree rule. We considered WASH variables associated with risk of severe cholera using a Cox proportional hazard regression model at *p*-value <0.2 using the training population. We verified that each selected independent variable fulfilled proportionality assumptions before inclusion into the model.

Construction of decision tree to develop composite WASH variable

We developed a composite rule for existing "Better" *versus* "Not Better" household WASH through a machine learning approach, recursive partitioning, to measure the association between WASH status and the incidence of severe cholera up to four years of follow-up. To create a single, binary composite WASH variable predicting the occurrence of severe cholera, we considered variables that were associated with severe cholera in bivariate analyses.[15]

Given relatively few endpoints compared to the total number of individuals followed, we accounted for the imbalanced case distribution during construction of the decision tree. The decision tree was designed by assuming a 1:670 loss function for the cost of false positive and false negative classification, and by defining 300 as the minimum number of observations required in each terminal node. The number of cross-validations was 10. We ran the algorithm with the training subpopulation and subsequently pruned the tree by the minimal complexity parameter, corresponding to a minimum error with at least two terminal nodes to determine the optimal decision rule for predicting severe cholera.

With the selected tree, a receiver operating characteristic (ROC) curve was constructed in the training population to find the optimal cut-off probability of the composite WASH variable for predicting severe cholera[16,17]. This threshold was used to create a composite WASH variable with two categories, one for "Better" household WASH associated with a lower probability of developing severe cholera in household members, and the other for "Not Better" household WASH, associated with a higher probability of developing severe cholera. We then evaluated the

For peer review only - http://bmjopen.bmj.com/site/about/guidelines.xhtml

dichotomous WASH variable in an independent validation population to confirm that the prediction rule exhibited similar sensitivity and specificity for severe cholera in both populations.

Protective association between WASH and severe cholera

Next, we measured the association between "Better" WASH household status and severe cholera in the entire population residing in non-intervention clusters. To evaluate this association, we analyzed the time from start of follow-up to the first severe cholera case using the Cox proportional hazard regression model.

The model was adjusted for potential confounding covariates, including age in years at start of follow-up, sex, and variables reflecting household socioeconomic status: monthly expenditure, house ownership, house having one room, and house wall constructed by brick/cement. We introduced variables into the model by mixed stepwise selection, using a combination of forward and backward selection with the cutoff p-value of 0.1 for both elimination and retention. Hazard ratios (HR) for severe cholera were estimated by exponentiating the coefficient for the composite WASH variable in models and protection was estimated as [(1- HR) X 100%] with 95% confidence intervals. Estimates were also adjusted for design effect of cluster randomization of the study clusters.

To determine cluster-level "Better" WASH coverage, the person-years of observation of household members living in "Better" WASH households in the cluster were divided by the person-years of the entire population in the same cluster. The association between cluster-level WASH coverage and incidence of severe cholera was assessed after adjustment for the same potential confounding variables in proportional hazard models using the same approach.

Statistical analyses were performed using R Studio analytical software for decision tree modeling (rpart package), tree plotting (rpart.plot package), and ROC curve illustration (pROC package). Other statistical analyses were performed using SAS version 9.4. All p-values were two-sided.

Results

For peer review only - http://bmjopen.bmj.com/site/about/guidelines.xhtml

Training and validation subpopulations

A total of 193,576 individuals in the non-intervention ICVB arm were included in the analysis, as shown in the CONSORT diagram in Figure 2. Of those, 80,720 individuals were present at baseline and 112,856 individuals were new entrants (107,381 in-migration individuals, 5,475 births). During the four-years of follow-up, a total of 292 severe cholera episodes were observed. The training set was composed of 96,943 individuals, 144 of whom developed severe cholera. The validation set was composed of 96,633 individuals, 148 of whom developed severe cholera. As shown in Table 1, baseline characteristics of the training and validation subpopulations were broadly comparable in terms of mean age, sex ratio, average monthly household expenditure, and other household characteristics.

Rule development for composite WASH variable predicting severe cholera

A bivariate analysis for each variable in the training population was performed to measure associations of individual WASH-related variables with the risk of severe cholera (Table 2). Drinking water source (51% reduction of risk; 95% CI: -20-80), distance to source of drinking water (39% reduction of risk ; 95% CI: 14-57) and drinking water treatment (36% reduction of risk; 95% CI: 11-54) were selected to determine the composite binary WASH variable. The resulting tree, shown in Figure 3, found that treatment of drinking water was the dominant bifurcation; regardless of other WASH variables in the tree, not treating drinking water categorized the household as having "Not better" WASH.

Performance of the composite WASH variable predicting severe cholera

In the training set, we found that an optimal cut-off value of 0.0012 for the composite WASH variable maximized the Youden index using the ROC curve, with an area under the curve (AUC) of 59% (95% CI: 55-63) (Figure 4). Under this threshold, the rule predicted 123 true positives of 144 severe cholera episodes, for a sensitivity of 85% (95% CI: 85-86), and 28,709 true negatives among 96,799 persons without severe cholera, yielding a specificity of 30% (95%

For peer review only - http://bmjopen.bmj.com/site/about/guidelines.xhtml

For peer review only

CI: 22-37). The composite WASH variable performed similarly when applied to the validation population, with a sensitivity of 82% (95% CI: 82-82) and specificity of 30% (95% CI: 22-37).

Prediction of severe cholera incidence by household WASH status in the total population

We applied the WASH prediction rule to the total population residing in the non-intervention clusters to predict severe cholera episode risk. 29.7% of households in the non-intervention arm were classified as having "Better" WASH and the remaining 70.3% were classified as having "Not Better" WASH.

The incidence of severe cholera in all age groups living in the "Better" WASH households was 57 per 100,000 person-years of observation (PYO), compared to 120 per 100,000 PYO in "Not Better" WASH households, a 47% (95% CI: 29-61, *p*-value *<0.001*) reduced risk of severe cholera, after adjusting for covariates. A protective association between living in a "Better" WASH households and severe cholera risk was seen in all age groups (Table 3). For individuals under the age of 15, living in a "Better" WASH households was associated with having 64% (95% CI: 36-80; *p*-value *<0.001*) reduced risk of severe cholera. The protective association of "Better" WASH household status was somewhat lower in the 15+ year age-group, where "Better" WASH was associated with a 43% (95% CI: 16-61; *p*-value: *0.004*) reduction in severe cholera risk.

There was a slight, non-significant negative association between rising proportion of "Better" WASH households in a cluster and incidence rate of severe cholera. In our model, for every ten percent increase in proportion of "Better" WASH households in a cluster, individuals living in that cluster experienced 4% lower risk of severe cholera, though this relationship was not significant (HR=0.996 (95% CI: 0.988-1.005; *p*-value*: 0.40*).

Discussion

Using a machine learning approach, we developed a composite WASH variable characterizing households at baseline that predicted the risk of severe cholera risk for four years

For peer review only

of follow-up of a cohort in a Dhaka slum. Water quality and access were the most significant factors associated with severe cholera risk.

This finding is consistent with previous literature and findings from Wolfe and colleagues' systematic review of case-control studies that found strong associations between household water quality and lowered incidence of cholera.[18] In this review of mostly epidemic cholera contexts, the authors found that eight WASH-related risk factors, including unimproved water source, untreated water, unsafe water storage and transport, were consistently associated with higher odds of cholera. As such, the importance of water practices in our determination of "Better" WASH is in line with the prevailing understanding of cholera risk. What is new about our findings is that the protective relationships between household WASH and cholera also pertain to cholera of life-threatening severity, are sustained for at least four years after initial characterization of household WASH at baseline, and pertain to a densely populated, poor slum in which cholera is highly endemic.

In our analysis, we found that individuals living in "Better" WASH households had 47% (95% CI: 29-61) reduced risk of severe cholera compared to individuals living in "Not Better" WASH households, after adjusting for age, gender, and socioeconomic factors. This statistically significant protective association was demonstrated in all age groups examined, though those under-15 exhibited a greater degree of protection compared to those over-15. This difference by age group may stem from the differences in WASH behaviors and exposure to cholera—wherein individuals 15 and older are more likely to have many exposures outside of the household at school, work, or play that could reduce the protective effects of living in a "Better" WASH home.

There are several strengths to our analysis that lend credibility to the findings. The analysis was evaluated in the context of a prospective CRT in a well-defined population with comprehensive follow-up for cholera detection. We followed this population for four-years, making it one of the longest evaluations of the relationship between household WASH and severe cholera incidence, and thereby shedding light on the long-term durability of WASH adaptations. Further, during the development of the composite WASH rule we reduced bias by

For peer review only - http://bmjopen.bmj.com/site/about/guidelines.xhtml

categorizing WASH variables without any prior knowledge of cholera incidence rate associated with each component category. We also rigorously validated the WASH prediction rule by using a validation sub-population to ensure that the rule was not over-fitted to the training sub-population.

Our analysis also has limitations. Firstly, household WASH variables were only evaluated once and applied to the whole study period. As household WASH variables were more likely to have improved rather than regressed over time due to the overall secular improvement in socioeconomic conditions in Bangladesh, this misclassification, which would have affected both households classified at baseline as having "Better" WASH and those classified as having "Not Better" WASH, would be expected to have led to more conservative estimates of protective associations with baseline WASH. Secondly, it should be noted that the household WASH variables included in this study were collected in the context of an OCV trial through a brief questionnaire designed to assess confounding variables in the evaluation of vaccine effectiveness. This approach may have led to loss of information, making our estimates of the WASH-severe cholera relationship conservative, and may also have identified variables that are "proxies" for actual water-related factors that directly mediate a reduced risk of severe cholera. Consequently, the WASH variables included in the composite rule may best be interpreted as *markers* of these direct mediators. Nonetheless, because these variables predicted severe cholera risk independently of non-WASH socioeconomic factors, the analysis underscores the importance of water-related WASH adaptations in determining the risk of severe cholera in this setting. Finally, it might be queried whether the relationship between "Better WASH" in the household and a lower risk of severe cholera might reflect the possibility that the "Better WASH" households instituted home-based and clinic-based care for cholera early, and thereby forestalled progression to severe cholera. We think that this was an unlikely explanation for our findings because the study population was highly sensitized to home- and clinic-based early treatment of watery diarrhea, the population had very good access to clinics and hospitals in the proximities of their homes, and all care for diarrhea at these facilities was low cost or free of charge. Moreover, in analyses to be published elsewhere, we found that the household WASH

For peer review only - http://bmjopen.bmj.com/site/about/guidelines.xhtml

prediction rule that we developed for severe cholera also strongly predicted the risk of all episodes of cholera seen at the treatment centers, regardless of severity.

Our findings indicate that there are existing culturally acceptable WASH improvements that may be impactful in controlling severe cholera in Mirpur, a dense slum population considered to have hyperendemic cholera. Past experiences in WASH intervention programs have shown that achieving sustainable and effective WASH interventions can be challenging. For example, an evaluation of a household water treatment and handwashing campaign in rural Guatemala found that three years after the intervention there were no differences in handwashing behavior, WASH conditions, or prevalence of childhood diarrhea in the community[19]. Other examples of poor long-term uptake and acceptability of WASH interventions are found in programs implemented in India, Zambia, and Kenya—all highlighting the difficulty of sustained behavior adoption.[20–22] In light of this, understanding *existing* "Better" WASH households and behaviors in endemic slum communities can provide valuable lessons on designing feasible and sustainable WASH interventions.

Recent studies have also found that Mirpur, Bangladesh, the site for this study, has very high rates of microbiological proliferation and contamination in the municipal water supply,[23] likely due to antiquated and now porous water and sewage pipes, making achievable WASH improvements paramount to improving community health. Our analysis found that there *are* existing household water adaptations in Mirpur associated with significantly lowered severe cholera risk, despite contaminated municipal water. The fact that the WASH adaptations practiced by 30% of the population were significantly predictive of lower severe cholera risk even within slum conditions speaks to the potential for existing knowledge to inform cholera control strategies.

Cholera transmission in urban slums may only intensify with the pressures of rapid urbanization combined with climate change effects. Endemic cholera in urban areas is a reality in Bangladesh that faces challenges that are different from previously studied epidemic and rural cholera transmission. Closely examining how some urban households have already made WASH

For peer review only - http://bmjopen.bmj.com/site/about/guidelines.xhtml

adaptations to reduce cholera risk may help design effective cholera control programs that are sustainable and achievable in similar settings.

Data Availability Statement

Data will be made available in a public, open access repository.

Ethics Statement

This study is a re-analysis of data from a cluster randomized trial entitled ‘Introduction of cholera vaccine in Bangladesh (ICVB)' conducted by the icddr,b in 2011. The ICVB trial received Institutional Review Board approval (icddr,b protocol number PR#17124) and Bangladesh National Research Ethics Committee approval. Participants gave informed consent to participate in the study before taking part.

Author Contributions

JDC conceptualized the study. SK and FC prepared the original draft. Data analysis was carried out by JP, FA, and DRK. Additionally JDC, FQ, FM, JHK, and AIK supervised the study. All authors participated in the writing of the manuscript and interpreting the data. All authors reviewed and approved the final manuscript.

Competing Interests

There are no competing interests from any of the authors.

Funding

This work was supported, in whole or in part, by the Bill & Melinda Gates Foundation [INV 025386]. Under the grant conditions of the Foundation, a Creative Commons Attribution 4.0 Generic License has already been assigned to the Author Accepted Manuscript version that might arise from this submission.

Acknowledgement

Icddr,b is grateful to the Governments of Bangladesh, Canada, Sweden and the UK for providing unrestricted support. Thanks to Dr. Nigel McMillan (Griffith University) for his scientific and

For peer review only

technical support in the manuscript. The funders had no role in study design, data collection and analysis, decision to publish, or preparation of the manuscript.

For peer review only

References

1. Ritchie, H. & Roser, M. Urbanization. *Our World Data* (2018).
2. Dhaka: fastest growing megacity in the world. *The World from PRX* https://www.pri.org/stories/2010-09-08/dhaka-fastest-growing-megacity-world.
3. Ross, A. G. P., Zaman, K. & Clemens, J. D. Health Concerns in Urban Slums: A Glimpse of Things to Come? *JAMA* **321**, 1973–1974 (2019).
4. Islam, M. T., Clemens, J. D. & Qadri, F. Cholera Control and Prevention in Bangladesh: An Evaluation of the Situation and Solutions. *J. Infect. Dis.* **218**, S171–S172 (2018).
5. Longini, I. M., Jr. *et al.* Epidemic and Endemic Cholera Trends over a 33-Year Period in Bangladesh. *J. Infect. Dis.* **186**, 246–251 (2002).
6. Asadgol, Z. *et al.* How climate change can affect cholera incidence and prevalence? A systematic review. *Environ. Sci. Pollut. Res.* **27**, 34906–34926 (2020).
7. Salerno, F., Gaetano, V. & Gianni, T. Urbanization and climate change impacts on surface water quality: Enhancing the resilience by reducing impervious surfaces. *Water Res.* **144**, 491–502 (2018).
8. Kumar, M., Deka, J. P. & Kumari, O. Development of Water Resilience Strategies in the context of climate change, and rapid urbanization: A discussion on vulnerability mitigation. *Groundw. Sustain. Dev.* **10**, 100308 (2020).

9. Taylor, D. L., Kahawita, T. M., Cairncross, S. & Ensink, J. H. J. The Impact of Water, Sanitation and Hygiene Interventions to Control Cholera: A Systematic Review. *PLOS ONE* **10**, e0135676 (2015).
10. George, C. M. *et al.* Randomized Controlled Trial of Hospital-Based Hygiene and Water Treatment Intervention (CHoBI7) to Reduce Cholera. *Emerg. Infect. Dis.* **22**, 233–241 (2016).
11. Luby, S. P., Davis, J., Brown, R. R., Gorelick, S. M. & Wong, T. H. F. Broad approaches to cholera control in Asia: Water, sanitation and handwashing. *Vaccine* **38 Suppl 1**, A110–A117 (2020).
12. Childs, L. *et al.* Evaluation of Knowledge and Practices Regarding Cholera, Water Treatment, Hygiene, and Sanitation Before and After an Oral Cholera Vaccination Campaign-Haiti, 2013-2014. *Am. J. Trop. Med. Hyg.* **95**, 1305–1313 (2016).
13. Qadri, F. *et al.* Feasibility and effectiveness of oral cholera vaccine in an urban endemic setting in Bangladesh: a cluster randomised open-label trial. *The Lancet* **386**, 1362–1371 (2015).
14. Khan, I. A. *et al.* Organization and implementation of an oral cholera vaccination campaign in an endemic urban setting in Dhaka, Bangladesh. *Glob. Health Action* **12**, (2019).
15. Strobl, C., Malley, J. & Tutz, G. An Introduction to Recursive Partitioning: Rationale, Application and Characteristics of Classification and Regression Trees, Bagging and Random Forests. *Psychol. Methods* **14**, 323–348 (2009).
16. Hand, D. J. Measuring classifier performance: a coherent alternative to the area under the ROC curve. *Mach. Learn.* **77**, 103–123 (2009).
17. Faraggi, D. & Reiser, B. Estimation of the area under the ROC curve. *Stat. Med.* **21**, 3093–3106 (2002).
18. Wolfe, M., Kaur, M., Yates, T., Woodin, M. & Lantagne, D. A Systematic Review and Meta-Analysis of the Association between Water, Sanitation, and Hygiene Exposures and Cholera in Case–Control Studies. *Am. J. Trop. Med. Hyg.* **99**, 534–545 (2018).
19. Arnold, B., Arana, B., Mäusezahl, D., Hubbard, A. & Colford, J. M., Jr. Evaluation of a pre-existing, 3-year household water treatment and handwashing intervention in rural Guatemala. *Int. J. Epidemiol.* **38**, 1651–1661 (2009).
20. Makutsa, P. *et al.* Challenges in Implementing a Point-of-Use Water Quality Intervention in Rural Kenya. *Am. J. Public Health* **91**, 1571–1573 (2001).

For peer review only

21. Firth, J. *et al.* Point-of-Use Interventions to Decrease Contamination of Drinking Water: A Randomized, Controlled Pilot Study on Efficacy, Effectiveness, and Acceptability of Closed Containers, Moringa oleifera, and In-home Chlorination in Rural South India. *Am. J. Trop. Med. Hyg.* **82**, 759–765 (2010).

22. Peletz, R. *et al.* Follow-Up Study to Assess the Use and Performance of Household Filters in Zambia. *Am. J. Trop. Med. Hyg.* **89**, 1190–1194 (2013).

23. Acharjee, M., Rahman, F., Jahan, F. & Noor, R. Bacterial Proliferation in Municipal Water Supplied in Mirpur Locality of Dhaka City, Bangladesh: Microbiological Study of Municipal Water. *CLEAN - Soil Air Water* **42**, 434–441 (2014).

For peer review only

For peer review only - http://bmjopen.bmj.com/site/about/guidelines.xhtml

Figure Legends

Figure 1. Map of ICVB study area and cholera surveillance treatment centers

Figure 2. CONSORT- Dynamic population during 4-year follow up period

Figure 3. Decision rule predicting severe cholera episode risk in the training subpopulation

Figure 4. ROC curve for the performance of the decision rule in the training subpopulation

Table 1. Baseline characteristics of the total, training, and validation subpopulations

	Total Population	Training sub-population	Validation sub-population
	n=193576	n=96943	n=96633
Age in years - mean (std)	22.9 (15.4)	22.9 (15.3)	22.9 (15.4)
Gender: Male - n (%)	94008 (48.6)	47365 (48.9)	46643 (48.3)
Monthly Expenditure† – mean (std)	10288.6 (5374.1)	10293.1 (4891.7)	10284.1 (5817.7)
Ownership: Own house - n (%)	28677 (14.8)	14549 (15.0)	14128 (14.6)
House: having one room - n (%)	165215 (85.3)	82427 (85.0)	82788 (85.7)
Wall: Brick/Cement - n (%)	139860 (72.3)	69880 (72.1)	69980 (72.4)

** std: standard deviation; n: number of individuals*
† expenditure in Bangladeshi takas (BDT)

Table 2: Bivariate relationship of WASH variables with severe cholera risk in the training subpopulation

**WASH Variable** *Criteria for Better WASH categorization*	Better WASH				Not Better WASH				Protective association between variable and severe cholera	
	n	Cases	Person-years	IR (95% CI) Per 100000/PY	n	Cases	Person-years	IR (95% CI) Per 100000/PY	Crude (95%CI)	*p*-value
**Shared toilet** *No*	4620	9	10253	88 (46, 169)	92323	135	134076	101 (85, 119)	21 (-55, 60)	0.49
**Toilet type** *Sanitary latrine with or without flush*	80974	122	118747	103 (86, 123)	15969	22	25582	86 (57, 131)	-24 (-95, 21)	0.36
**Drinking Water Source:** *Own tap/well/pump/bottled or vendor water*	4187	5	8993	56 (23, 134)	92756	139	135337	103 (87, 121)	51 (-20, 80)	0.12
**Water availability:** *Tap/tube well/well water is available all the time*	67831	95	93293	102 (83, 125)	29112	49	51036	96 (73, 127)	-15 (-64, 19)	0.43
**Distance to source of drinking water** *Using median as cut off*	43806	47	64461	73 (55, 97)	53137	97	79869	121 (100, 148)	39 (14, 57)	<0.01

For peer review only - http://bmjopen.bmj.com/site/about/guidelines.xhtml

Drinking Water Treatment: *Filtered/Boiled/chemical treated*	54275	60	77380	78 (60, 100)	42668	84	66950	125 (101, 155)	36 (11, 54)	<0.01
Waste disposal location: *Fixed*	80198	109	116056	94 (78, 113)	16745	35	28273	124 (89, 172)	20 (-17, 46)	0.25
Hand Washing Water Available § *Yes*	93758	138	138159	100 (85, 118)	3185	6	6170	97 (44, 216)	-11 (-152, 51)	0.8
Hand Washing Soap Available § *Yes*	91514	133	135316	98 (83, 117)	5429	11	9014	122 (68, 220)	17 (-54, 55)	0.56
Shared kitchen *No*	86713	118	120182	98 (82, 118)	10230	26	24147	108 (73, 158)	-5 (-62, 32)	0.83

** IR: incidence rate; n: number of individuals*

§ Indicates WASH condition observed directly by study team

For peer review only

Table 3. Protective association between "Better" WASH and severe cholera risk in the total study population

Age at start of follow-up	"Better" WASH				"Not Better" WASH				Protective association between "Better" WASH and severe cholera			
	n	Cases	Person-years	IR per 100000/PYs (95% CI)	N	Cases	Person-years	IR per 100000/PYs (95% CI)	Crude (95%CI)	*p*-value	Adjusted* (95%CI)	*p*-value
< 5 years	7552	5	10518	48 (20, 114)	17149	42	24366	172 (127, 233)	72 (25, 89)	0.012	69 (15, 88)	0.022
5-14 years	8861	4	14618	27 (10, 73)	25318	32	42389	75 (53, 107)	63 (6, 85)	0.037	63 (6, 85)	0.037
15+ years	40983	39	59680	65 (48, 89)	93713	170	136945	124 (107, 144)	46 (20, 64)	0.002	43 (16, 61)	0.004
All	57396	48	84817	57 (43, 75)	136180	244	203701	120 (106, 136)	52 (34, 65)	<0.001	47 (29, 61)	<0.001

** IR: incidence rate; n: number of individuals*

** Adjusted for design effect and selected covariates by stepwise selection using cutoff 0.1 for both of elimination and addition; For entire population, selected covariates were age, gender, home ownership and house having one room; For <5 years, selected covariate was home ownership; For 5-14 years, no covariate was selected; For 15+ years, selected covariates were age, gender and house having one room*

For peer review only - http://bmjopen.bmj.com/site/about/guidelines.xhtml

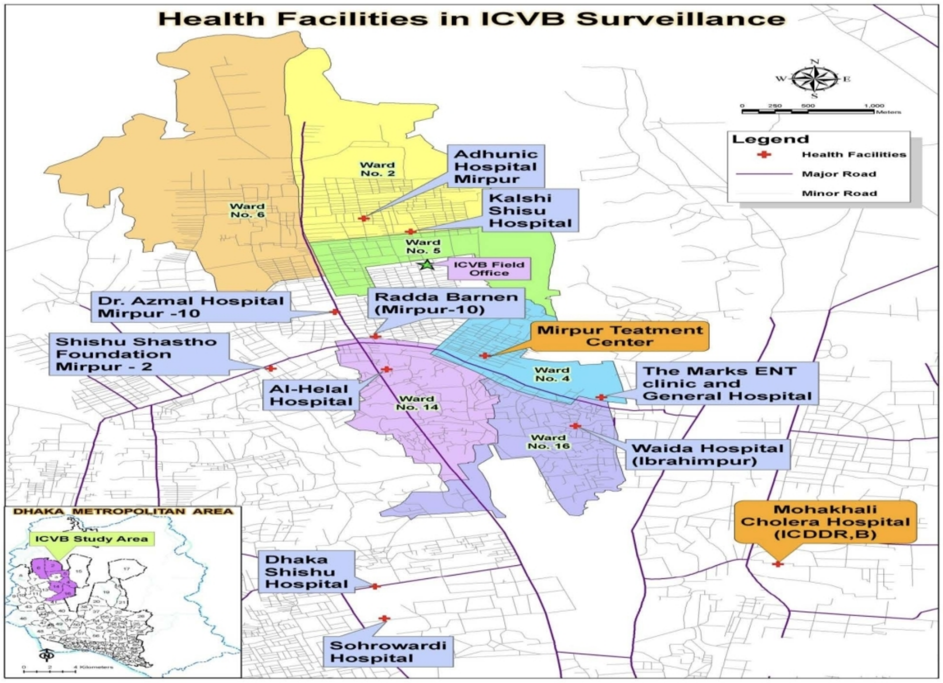

Figure 1. Map of ICVB study area and cholera surveillance treatment centers

221x177mm (237 x 211 DPI)

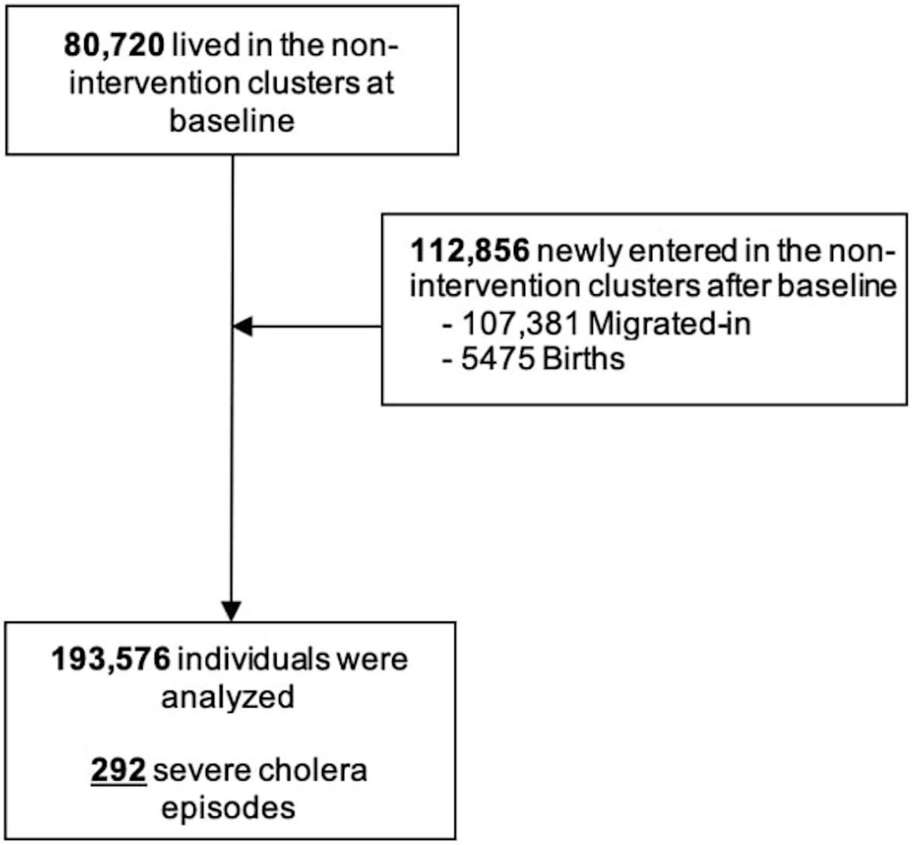

Figure 2. CONSORT- Dynamic population during 4-year follow up period

109x102mm (300 x 300 DPI)

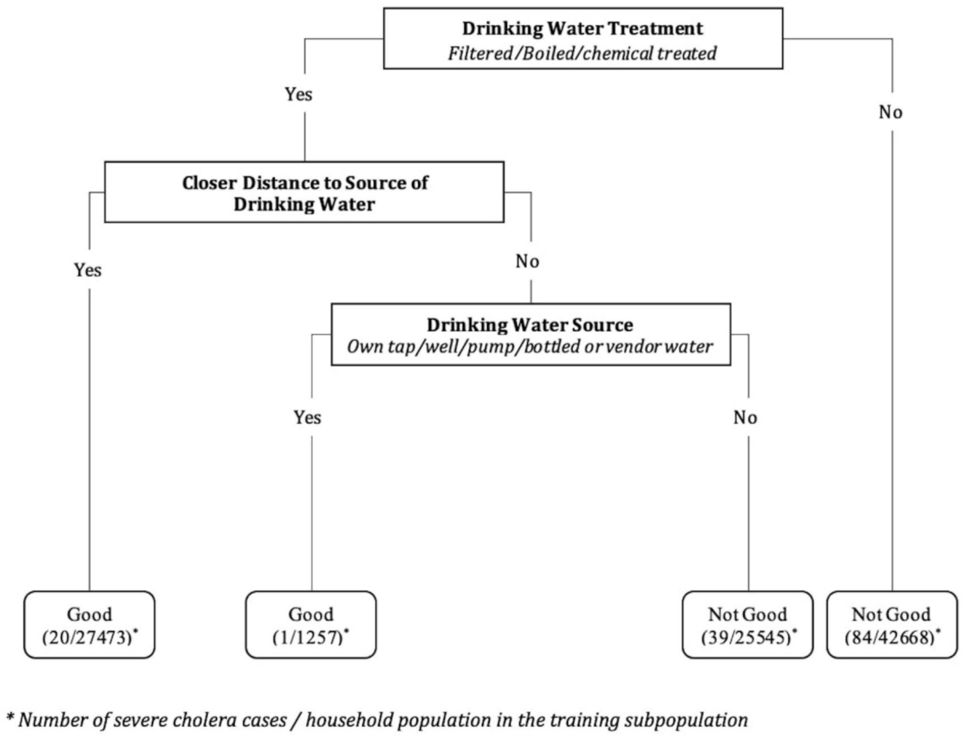

Figure 3. Decision rule predicting severe cholera episode risk in the training subpopulation

150x115mm (300 x 300 DPI)

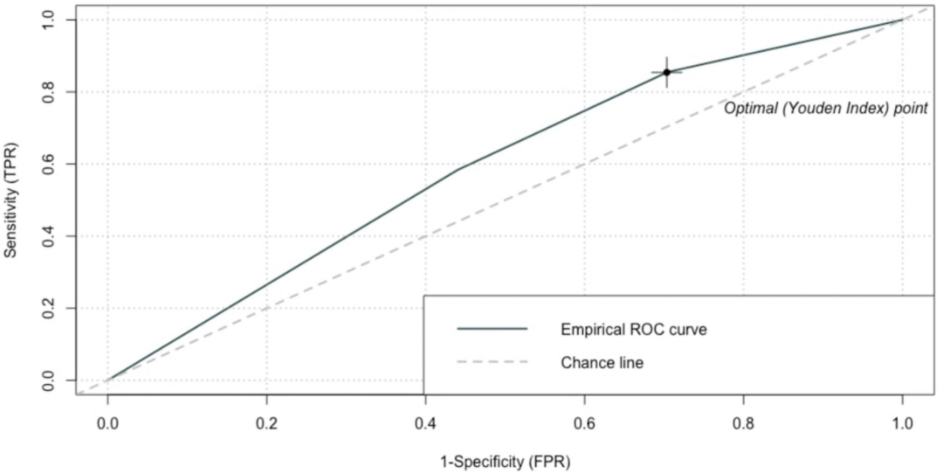

AUC = 59% (95% CI, 55-63); optimal cut-off probability using the Youden index = 0.0012

Figure 4. ROC curve for the performance of the decision rule in the training subpopulation

180x100mm (300 x 300 DPI)

STROBE Statement—Checklist of items that should be included in reports of ***cohort studies***

	Item No	Recommendation	Page No
**Title and abstract**	1	(*a*) Indicate the study's design with a commonly used term in the title or the abstract	1
		(*b*) Provide in the abstract an informative and balanced summary of what was done and what was found	2
**Introduction**			
Background/rationale	2	Explain the scientific background and rationale for the investigation being reported	4
Objectives	3	State specific objectives, including any prespecified hypotheses	4
**Methods**			
Study design	4	Present key elements of study design early in the paper	1-2
Setting	5	Describe the setting, locations, and relevant dates, including periods of recruitment, exposure, follow-up, and data collection	2, 4-5
Participants	6	(*a*) Give the eligibility criteria, and the sources and methods of selection of participants. Describe methods of follow-up	5-6
		(*b*) For matched studies, give matching criteria and number of exposed and unexposed	N/A
Variables	7	Clearly define all outcomes, exposures, predictors, potential confounders, and effect modifiers. Give diagnostic criteria, if applicable	5-6
Data sources/ measurement	8*	For each variable of interest, give sources of data and details of methods of assessment (measurement). Describe comparability of assessment methods if there is more than one group	5-7
Bias	9	Describe any efforts to address potential sources of bias	6-7
Study size	10	Explain how the study size was arrived at	N/A
Quantitative variables	11	Explain how quantitative variables were handled in the analyses. If applicable, describe which groupings were chosen and why	6-7
Statistical methods	12	(*a*) Describe all statistical methods, including those used to control for confounding	6-8
		(*b*) Describe any methods used to examine subgroups and interactions	6-8
		(*c*) Explain how missing data were addressed	N/A
		(*d*) If applicable, explain how loss to follow-up was addressed	6
		(*e*) Describe any sensitivity analyses	N/A
**Results**			
Participants	13*	(a) Report numbers of individuals at each stage of study—eg numbers potentially eligible, examined for eligibility, confirmed eligible, included in the study, completing follow-up, and analysed	8
		(b) Give reasons for non-participation at each stage	N/A
		(c) Consider use of a flow diagram	12
Descriptive data	14*	(a) Give characteristics of study participants (eg demographic, clinical, social) and information on exposures and potential confounders	13
		(b) Indicate number of participants with missing data for each variable of interest	N/A
		(c) Summarise follow-up time (eg, average and total amount)	8
Outcome data	15*	Report numbers of outcome events or summary measures over time	8

For peer review only

Main results	16	(*a*) Give unadjusted estimates and, if applicable, confounder-adjusted estimates and their precision (eg, 95% confidence interval). Make clear which confounders were adjusted for and why they were included	9
		(*b*) Report category boundaries when continuous variables were categorized	N/A
		(*c*) If relevant, consider translating estimates of relative risk into absolute risk for a meaningful time period	N/A
Other analyses	17	Report other analyses done—eg analyses of subgroups and interactions, and sensitivity analyses	11
**Discussion**			
Key results	18	Summarise key results with reference to study objectives	10
Limitations	19	Discuss limitations of the study, taking into account sources of potential bias or imprecision. Discuss both direction and magnitude of any potential bias	12
Interpretation	20	Give a cautious overall interpretation of results considering objectives, limitations, multiplicity of analyses, results from similar studies, and other relevant evidence	11-13
Generalisability	21	Discuss the generalisability (external validity) of the study results	12-13
**Other information**			
Funding	22	Give the source of funding and the role of the funders for the present study and, if applicable, for the original study on which the present article is based	13

*Give information separately for exposed and unexposed groups.

Note: An Explanation and Elaboration article discusses each checklist item and gives methodological background and published examples of transparent reporting. The STROBE checklist is best used in conjunction with this article (freely available on the Web sites of PLoS Medicine at http://www.plosmedicine.org/, Annals of Internal Medicine at http://www.annals.org/, and Epidemiology at http://www.epidem.com/). Information on the STROBE Initiative is available at http://www.strobe-statement.org.

For peer review only

BMJ Open

Are better existing WASH practices in urban slums associated with a lower long-term risk of severe cholera? A prospective cohort study with four years of follow-up in Mirpur, Bangladesh

Journal:	*BMJ Open*
Manuscript ID	bmjopen-2022-060858.R2
Article Type:	Original research
Date Submitted by the Author:	23-Aug-2022
Complete List of Authors:	Kang, Sophie; International Vaccine Institute, Epidemiology, Public Health, and Impact Unit<br>Chowdhury, Fahima; International Centre for Diarrhoeal Disease Research Bangladesh; Griffith University Menzies Health Institute Queensland<br>Park, Juyeon; International Vaccine Institute, Epidemiology, Public Health, and Impact Unit; University of Cambridge, Department of Medicine<br>Ahmed, Tasnuva; International Centre for Diarrhoeal Disease Research Bangladesh<br>Tadesse, Birkneh Tilahun; International Vaccine Institute, Epidemiology, Public Health, and Impact Unit<br>Islam, Md. Taufiqul; International Centre for Diarrhoeal Disease Research Bangladesh<br>Kim, Deok Ryun; International Vaccine Institute, Epidemiology, Public Health, and Impact Unit<br>Im, Justin; International Vaccine Institute<br>Aziz, Asma; International Vaccine Institute, Epidemiology, Public Health, and Impact Unit<br>Hoque, Masuma; International Centre for Diarrhoeal Disease Research Bangladesh<br>Pak, GiDeok; International Vaccine Institute<br>Khanam, Farhana; International Centre for Diarrhoeal Disease Research Bangladesh<br>Ahmmed, Faisal; International Centre for Diarrhoeal Disease Research Bangladesh<br>Liu , Xinxue ; University of Oxford Oxford Vaccine Group<br>Zaman, K; International Centre for Diarrhoeal Disease Research Bangladesh<br>Khan, Ashraful Islam; International Centre for Diarrhoeal Disease Research<br>Kim, Jerome ; International Vaccine Institute<br>Marks, Florian ; International Vaccine Institute; University of Cambridge, Department of Medicine<br>Qadri, Firdausi ; International Centre for Diarrhoeal Disease Research Bangladesh<br>Clemens, John; International Vaccine Institute, Epidemiology, Public Health, and Impact Unit; International Centre for Diarrhoeal Disease

	Research Bangladesh
**Primary Subject Heading**:	Global health
Secondary Subject Heading:	Epidemiology, Infectious diseases, Public health
Keywords:	EPIDEMIOLOGY, Epidemiology < INFECTIOUS DISEASES, INFECTIOUS DISEASES, PUBLIC HEALTH

I, the Submitting Author has the right to grant and does grant on behalf of all authors of the Work (as defined in the below author licence), an exclusive licence and/or a non-exclusive licence for contributions from authors who are: i) UK Crown employees; ii) where BMJ has agreed a CC-BY licence shall apply, and/or iii) in accordance with the terms applicable for US Federal Government officers or employees acting as part of their official duties; on a worldwide, perpetual, irrevocable, royalty-free basis to BMJ Publishing Group Ltd ("BMJ") its licensees and where the relevant Journal is co-owned by BMJ to the co-owners of the Journal, to publish the Work in this journal and any other BMJ products and to exploit all rights, as set out in our licence.

The Submitting Author accepts and understands that any supply made under these terms is made by BMJ to the Submitting Author unless you are acting as an employee on behalf of your employer or a postgraduate student of an affiliated institution which is paying any applicable article publishing charge ("APC") for Open Access articles. Where the Submitting Author wishes to make the Work available on an Open Access basis (and intends to pay the relevant APC), the terms of reuse of such Open Access shall be governed by a Creative Commons licence – details of these licences and which Creative Commons licence will apply to this Work are set out in our licence referred to above.

Other than as permitted in any relevant BMJ Author's Self Archiving Policies, I confirm this Work has not been accepted for publication elsewhere, is not being considered for publication elsewhere and does not duplicate material already published. I confirm all authors consent to publication of this Work and authorise the granting of this licence.

For peer review only

Are better existing WASH practices in urban slums associated with a lower long-term risk of severe cholera? A prospective cohort study with four years of follow-up in Mirpur, Bangladesh

Sophie Kang[1*], Fahima Chowdhury[2,7*], Juyeon Park[1,4], Tasnuva Ahmed[2], Birkneh Tilahun Tadesse[1], Md. Taufiqul Islam[2], Deok Ryun Kim[1], Justin Im[1], Asma Binte Aziz[1], Masuma Hoque[2], Gideok Pak[1], Farhana Khanam[2], Faisal Ahmmed[2], Xinxue Liu[3], Khalequ Zaman[2], Ashraful Islam Khan[2], Jerome H. Kim[1], Florian Marks[1,4,5], Firdausi Qadri[2], John D. Clemens[1,2,6]

1 International Vaccine Institute, Seoul, Republic of Korea

2 International Centre for Diarrheal Disease Research, Bangladesh, Dhaka, Bangladesh

3 Oxford Vaccine Group, Department of Pediatrics, University of Oxford, Oxford OX3 9DU, UK

4 Department of Medicine, University of Cambridge, Cambridge, United Kingdom

5 University of Antananarivo, Antananarivo, Madagascar

6 UCLA Fielding School of Public Health, Los Angeles, CA 90095-1772, USA

7 Menzies Health Institute Queensland, Griffith University, Gold Coast, Australia

* Contributed equally

Correspondence to Sophie Kang, International Vaccine Institute; Sophie.kang@ivi.int

For peer review only

Abstract

Objective

To investigate the association between existing household WASH practices and severe cholera risk in a dense urban slum where cholera is highly endemic.

Design, Setting, and Participants

We assembled a large prospective cohort within a cluster randomized trial evaluating the effectiveness of oral cholera vaccine. Our dynamic cohort population (n=193,576) comprised individuals living in the "non-intervention" clusters of the trial, and were followed over four years. This study was conducted in a dense urban slum community of Dhaka, Bangladesh and cholera surveillance was undertaken in twelve hospitals serving the study area.

Primary outcome measure

First severe cholera episode detected during follow-up period.

Methods

For peer review only

We applied a machine learning algorithm on a training subpopulation (n=96,943) to develop a binary ("Better", "Not Better") composite WASH variable predictive of severe cholera. The WASH rule was evaluated for performance in a separate validation subpopulation (n=96,633). Afterwards, we used Cox-regression models to evaluate the association between "Better" WASH households and severe cholera risk over 4 years in the entire study population.

Results

The "Better" WASH rule found that water quality and access were the most significant factors associated with severe cholera risk. Members of "Better" WASH households, constituting one-third of the population, had a 47% reduced risk of severe cholera (95% CI: 29-69; *p*-value <0.001), after adjusting for covariates. The protective association between living in a "Better" WASH household and severe cholera persisted in all age groups.

Conclusions

Salutary existing household WASH practices were associated with a significantly reduced long-term risk of severe cholera in an urban slum of Dhaka. These findings suggest that WASH adaptations already practiced in the community may be important for developing and implementing effective and sustainable cholera control programs in similar settings.

Strengths and limitations of this study

For peer review only

- We studied a cohort prospectively followed for four years, making this analysis one of the longest uninterrupted evaluations of the relationship between household WASH and endemic cholera.
- The multivariable WASH prediction rule was rigorously validated using a separate sub-population and thus avoided overfitting to the training set.
- We focused on the existing variability of household WASH within a dense slum community to demonstrate that there are salutary practices that may reduce the long-term risk of severe cholera.
- Household WASH factors were only evaluated once, either at baseline or when participants entered the study area, and this status applied to the entire follow-up period .
- The household WASH variables included in this study were collected in the context of an OCV trial and not optimized for describing WASH factors independently.

For peer review only

Introduction

The developing world has seen a rapid expansion of urban areas due, in part, to influxes into squalid urban slums. With an estimated 55% of the world's population living in urban areas, 1-in-3 of those urban dwellers live in slum households [1]. Slum households are defined as those which lack one or more of the following conditions: access to improved water, access to improved sanitation, sufficient living area, and durability of housing.

In Bangladesh urban dwellers currently account for 38% of the population and are expected to exceed 50% by 2030; furthermore, 47% of the urban population lives in slums, where residents are at increased risk for waterborne diseases, including cholera [2,3]. Cholera is a major cause of morbidity and mortality in Bangladesh [4]. Previously thought to be a rural disease in Bangladesh, cholera is now becoming a disease of cities and slums where living conditions create different challenges for cholera control from those encountered in rural settings [5]. Lessons learned from rural areas, and particularly in epidemic situations, may not be applicable to the changing pattern of cholera endemicity in urban areas. Specific studies on how endemic cholera can be controlled in these urban slums are needed.

Exacerbating the urban cholera situation further is the role of climate change. Rising temperatures and increased precipitation associated with climate change are significant

For peer review only - http://bmjopen.bmj.com/site/about/guidelines.xhtml

predictors of cholera incidence, with strong evidence in studies of Bangladesh where warmer, wetter conditions are associated with major cholera outbreaks [6]. Climate change effects in urban areas have added negative implications for water quality, and studies have found that increased rainfall intensity combined with impervious urban surfaces are significant predictors of sewer overflows that greatly impact water quality [7,8].

Improvements of water quality, sanitation, and hygiene (WASH) and oral cholera vaccine (OCV) are the major tools for the prevention of endemic cholera, including in urban slums. However, while WASH interventions are frequently employed to control cholera, evidence regarding their effectiveness is inconsistent and successful implementation may be stymied by limited cultural acceptability, low uptake, and poor community acceptance [9, 10, 11, 12]. Our cluster, randomized trial (CRT) of OCV and WASH in an urban Dhaka slum, which failed to demonstrate that WASH added to protection against severe cholera by OCV, is illustrative [13].

In this re-analysis of the CRT, we followed households in the 'non-intervention' arm to investigate how existing WASH practices and adaptations in the slums may be associated with lower severe cholera risk. We assessed long-term severe cholera risk over four years and sought to identify the salutary practices that might inform the development of future effective, acceptable, and sustainable WASH interventions in cholera-endemic urban populations.

Methods

Trial and Population

In 2011, the International Centre for Diarrheal Disease Research, Bangladesh (icddr,b) conducted a cluster randomized control trial entitled 'Introduction of cholera vaccine in Bangladesh (ICVB)' [13] in six selected wards of Mirpur, Dhaka to evaluate the feasibility and effectiveness of oral cholera vaccine (OCV), both deployed alone and in conjunction with WASH interventions. Vaccination with Shanchol, a two-dose OCV was carried out between 17 February and 6 April, 2011.

Households were grouped into 90 geographic clusters with an average population of 2988 households per cluster (ranging from 2288 to 4299 households per cluster). Clusters were

For peer review only - http://bmjopen.bmj.com/site/about/guidelines.xhtml

randomly assigned (1:1:1) to one of three arms: a two-dose regimen of OCV alone, OCV with a WASH intervention, or no intervention (control) [13]. The two doses of OCV were administered at a 14-day interval. The WASH intervention consisted of a behavioral change intervention which focused on use of a household handwashing station and a chlorine dispenser for the treatment of household drinking water[13]. Healthy, non-pregnant individuals aged one year or older were eligible for vaccination in this cluster randomized trial (CRT), and each cluster was separated from the adjoining cluster by at least a 30-meter buffer area to minimize diffusion of the WASH messages between clusters.

For peer review only

Demographic Surveillance

The study population was characterized with a baseline demographic census and recurring census updates to surveille births, deaths, and migrations in the community. The baseline demographic census was conducted before the start of the ICVB vaccination campaign and updated bi-annually. Verbal consent for participation in the surveillance was obtained and documented in a questionnaire at the time of the baseline census and at each bi-annual update. A "household" was defined as persons sharing the same cooking pot.

In addition to the basic demographic information, household-level socioeconomic status, WASH data, and geographical locations of each household were collected during the baseline census. For households that were not present at the baseline census, WASH characteristics were assessed at the first bi-annual census update they were captured in. Household WASH characteristics were not re-assessed for new births in a household already characterized during the study period. All individuals living in the study area were provided with a study identification card containing the unique participant identification (PID) number that was recorded in computerized study databases.

Disease Surveillance

Surveillance for cholera was conducted between April 2011 to November 2015 at two icddr,b hospitals and ten hospitals serving the study area shown in Figure 1. Study surveillance

For peer review only - http://bmjopen.bmj.com/site/about/guidelines.xhtml

staff were present at each health facility throughout the day to facilitate reporting of diarrheal cases from the study area. Patients from the study area were identified in the treatment centers with their study identification card or by searching their identities in on-site computerized census database. Clinical examination was carried out by physicians, and designated study staff completed data forms and obtained fecal specimens after obtaining written informed consent.

A diarrheal visit was defined as having 3 or more loose stools or, 1-2 or an indeterminate number of loose stools with evidence of dehydration, in the 24 hours before presentation[13]. If the date of discharge from an earlier diarrheal visit and the date of symptom onset for the subsequent diarrheal visit were within 7 days of one another, then both visits were considered part of the same diarrheal episode. The onset of a diarrheal episode was defined as starting on the day the patient first reported loose or liquid stools.

Fecal samples were examined for *V. cholerae* O1 or O139 serogroups, biotype, and Inaba and Ogawa serotypes using conventional methods[13]. A cholera episode was defined as a diarrheal episode in which a fecal specimen yielded *V. cholerae* O1 or O139, with no passage of bloody stools during the episode. Severely dehydrating cholera was defined by the presence of at least two of the following symptoms or signs of severe dehydration: sunken eyes, dry tongue, thirst, irritable condition, less active than usual along with inability to drink, skin pinch goes back slowly, and low volume of radial pulse[13]. A severe cholera episode was one in which the patient exhibited severe dehydration during any visit of the episode. The primary outcome in this analysis was the first severe cholera episode detected during follow-up.

<u>Patient and Public Involvement</u>

This analysis utilizes data that originates from the ICVB cluster randomized trial conducted by the icddr,b in 2011. Given that ten years have passed since the original study, the participants were not directly involved in developing or informing the design of the analysis described in this paper. That said, the original ICVB trial involved strong social mobilization and community engagement to improve the conduct of the study.

For peer review only - http://bmjopen.bmj.com/site/about/guidelines.xhtml

The research questions addressed in the ICVB cluster randomized trial were developed due to the pressing need to understand the impact of oral cholera vaccines in urban Bangladesh. Advocacy meetings with local government representatives, pediatric associations, and NGOs were held in order to inform the design and conduct of the ICVB trial.

Analytical Approach

Population Under Follow-up

We considered a dynamic population for this analysis, which included the population present in the non-intervention arm at baseline and new entrants into the non-intervention study area during the study period. For those present at baseline, the start of follow-up was defined as the median date of first Shanchol dose in the nearest intervention cluster. For new residents, the start of follow up was defined as either the date of birth or the date of migration into the study area. The end of follow-up was defined as either the end of surveillance, 4-years after baseline; date of death; date of migration out of the cluster; or onset date of first severe cholera episode, whichever came first. Person-years of observation (PYO) were calculated from the sum of follow up periods for individuals under analysis.

Selection of WASH variables for analyses

We first examined the ten household WASH variables ascertained in the demographic censuses and categorized each variable (shared toilet, drinking water source, distance to source of drinking water, drinking water treatment, toilet type, water availability, waste disposal location, hand washing water available, hand washing soap available, and shared kitchen into two categories: "Better" versus "Not Better". The categorization of WASH variables was based on local context-informed judgement and the distribution of the study population into categories of the WASH variables, but without prior information on cholera incidence rates associated with each variable category.

We randomly divided the population of clusters of the "non-intervention arm" of the trial into two subpopulations– 50% of the households into a "training" population and the other 50%

For peer review only - http://bmjopen.bmj.com/site/about/guidelines.xhtml

into a "validation" population. The training population was used to develop a recursive portioning tree to define the composite WASH variable, and the validation population was subsequently used to cross-validate the decision tree rule. We considered WASH variables associated with risk of severe cholera using a Cox proportional hazard regression model at *p*-value <0.2 using the training population. We verified that each selected independent variable fulfilled proportionality assumptions before inclusion into the model.

Construction of decision tree to develop composite WASH variable

We developed a composite rule for existing "Better" *versus* "Not Better" household WASH through a machine learning approach, recursive partitioning, to measure the association between WASH status and the incidence of severe cholera up to four years of follow-up. To create a single, binary composite WASH variable predicting the occurrence of severe cholera, we considered variables that were associated with severe cholera in bivariate analyses.[14]

Given relatively few endpoints compared to the total number of individuals followed, we accounted for the imbalanced case distribution during construction of the decision tree. The decision tree was designed by assuming a 1:670 loss function for the cost of false positive and false negative classification, and by defining 300 as the minimum number of observations required in each terminal node. The number of cross-validations was 10. We ran the algorithm with the training subpopulation and subsequently pruned the tree by the minimal complexity parameter, corresponding to a minimum error with at least two terminal nodes to determine the optimal decision rule for predicting severe cholera.

With the selected tree, a receiver operating characteristic (ROC) curve was constructed in the training population to find the optimal cut-off probability of the composite WASH variable for predicting severe cholera[15,16]. This threshold was used to create a composite WASH variable with two categories, one for "Better" household WASH associated with a lower probability of developing severe cholera in household members, and the other for "Not Better" household WASH, associated with a higher probability of developing severe cholera. We then evaluated the

For peer review only - http://bmjopen.bmj.com/site/about/guidelines.xhtml

dichotomous WASH variable in an independent validation population to confirm that the prediction rule exhibited similar sensitivity and specificity for severe cholera in both populations.

Protective association between WASH and severe cholera

Next, we measured the association between "Better" WASH household status and severe cholera in the entire population residing in non-intervention clusters. To evaluate this association, we analyzed the time from start of follow-up to the first severe cholera case using the Cox proportional hazard regression model.

The model was adjusted for potential confounding covariates, including age in years at start of follow-up, sex, and variables reflecting household socioeconomic status: monthly expenditure, house ownership, house having one room, and house wall constructed by brick/cement. We introduced variables into the model by mixed stepwise selection, using a combination of forward and backward selection with the cutoff *p*-value of 0.1 for both elimination and retention. Hazard ratios (HR) for severe cholera were estimated by exponentiating the coefficient for the composite WASH variable in models and protection was estimated as [(1- HR) X 100%] with 95% confidence intervals. Estimates were also adjusted for design effect of cluster randomization of the study clusters.

To determine cluster-level "Better" WASH coverage, the person-years of observation of household members living in "Better" WASH households in the cluster were divided by the person-years of the entire population in the same cluster. The association between cluster-level WASH coverage and incidence of severe cholera was assessed after adjustment for the same potential confounding variables in proportional hazard models using the same approach.

Statistical analyses were performed using R Studio analytical software for decision tree modeling (rpart package), tree plotting (rpart.plot package), and ROC curve illustration (pROC package). Other statistical analyses were performed using SAS version 9.4. All *p*-values were two-sided.

Results

For peer review only - http://bmjopen.bmj.com/site/about/guidelines.xhtml

Training and validation subpopulations

A total of 193,576 individuals in the non-intervention ICVB arm were included in the analysis, as shown in the CONSORT diagram in Figure 2. Of those, 80,720 individuals were present at baseline and 112,856 individuals were new entrants (107,381 in-migration individuals, 5,475 births). During the four-years of follow-up, a total of 292 severe cholera episodes were observed. The training set was composed of 96,943 individuals, 144 of whom developed severe cholera. The validation set was composed of 96,633 individuals, 148 of whom developed severe cholera. As shown in Table 1, baseline characteristics of the training and validation subpopulations were broadly comparable in terms of mean age, sex ratio, average monthly household expenditure, and other household characteristics.

Rule development for composite WASH variable predicting severe cholera

A bivariate analysis for each variable in the training population was performed to measure associations of individual WASH-related variables with the risk of severe cholera (Table 2). Drinking water source (51% reduction of risk; 95% CI: -20-80), distance to source of drinking water (39% reduction of risk ; 95% CI: 14-57) and drinking water treatment (36% reduction of risk; 95% CI: 11-54) were selected to determine the composite binary WASH variable. The resulting tree, shown in Figure 3, found that treatment of drinking water was the dominant bifurcation; regardless of other WASH variables in the tree, not treating drinking water categorized the household as having "Not better" WASH.

Performance of the composite WASH variable predicting severe cholera

In the training set, we found that an optimal cut-off value of 0.0012 for the composite WASH variable maximized the Youden index using the ROC curve, with an area under the curve (AUC) of 59% (95% CI: 55-63) (Figure 4). Under this threshold, the rule predicted 123 true positives of 144 severe cholera episodes, for a sensitivity of 85% (95% CI: 85-86), and 28,709 true negatives among 96,799 persons without severe cholera, yielding a specificity of 30% (95%

For peer review only - http://bmjopen.bmj.com/site/about/guidelines.xhtml

CI: 22-37). The composite WASH variable performed similarly when applied to the validation population, with a sensitivity of 82% (95% CI: 82-82) and specificity of 30% (95% CI: 22-37).

Prediction of severe cholera incidence by household WASH status in the total population

We applied the WASH prediction rule to the total population residing in the non-intervention clusters to predict severe cholera episode risk. 29.7% of households in the non-intervention arm were classified as having "Better" WASH and the remaining 70.3% were classified as having "Not Better" WASH.

The incidence of severe cholera in all age groups living in the "Better" WASH households was 57 per 100,000 person-years of observation (PYO), compared to 120 per 100,000 PYO in "Not Better" WASH households, a 47% (95% CI: 29-61, *p*-value *<0.001*) reduced risk of severe cholera, after adjusting for covariates. A protective association between living in a "Better" WASH households and severe cholera risk was seen in all age groups (Table 3). For individuals under the age of 15, living in a "Better" WASH households was associated with having 64% (95% CI: 36-80; *p*-value *<0.001*) reduced risk of severe cholera. The protective association of "Better" WASH household status was somewhat lower in the 15+ year age-group, where "Better" WASH was associated with a 43% (95% CI: 16-61; *p*-value: *0.004*) reduction in severe cholera risk.

There was a slight, non-significant negative association between rising proportion of "Better" WASH households in a cluster and incidence rate of severe cholera. In our model, for every ten percent increase in proportion of "Better" WASH households in a cluster, individuals living in that cluster experienced 4% lower risk of severe cholera, though this relationship was not significant (HR=0.996 (95% CI: 0.988-1.005; *p*-value*: 0.40*).

Discussion

Using a machine learning approach, we developed a composite WASH variable characterizing households at baseline that predicted the risk of severe cholera risk for four years

For peer review only

of follow-up of a cohort in a Dhaka slum. Water quality and access were the most significant factors associated with severe cholera risk.

This finding is consistent with previous literature and findings from Wolfe and colleagues' systematic review of case-control studies that found strong associations between household water quality and lowered incidence of cholera.[17] In this review of mostly epidemic cholera contexts, the authors found that eight WASH-related risk factors, including unimproved water source, untreated water, unsafe water storage and transport, were consistently associated with higher odds of cholera. As such, the importance of water practices in our determination of "Better" WASH is in line with the prevailing understanding of cholera risk. What is new about our findings is that the protective relationships between household WASH and cholera also pertain to cholera of life-threatening severity, are sustained for at least four years after initial characterization of household WASH at baseline, and pertain to a densely populated, poor slum in which cholera is highly endemic.

In our analysis, we found that individuals living in "Better" WASH households had 47% (95% CI: 29-61) reduced risk of severe cholera compared to individuals living in "Not Better" WASH households, after adjusting for age, gender, and socioeconomic factors. This statistically significant protective association was demonstrated in all age groups examined, though those under-15 exhibited a greater degree of protection compared to those over-15. This difference by age group may stem from the differences in WASH behaviors and exposure to cholera—wherein individuals 15 and older are more likely to have many exposures outside of the household at school, work, or play that could reduce the protective effects of living in a "Better" WASH home.

There are several strengths to our analysis that lend credibility to the findings. The analysis was evaluated in the context of a prospective CRT in a well-defined population with comprehensive follow-up for cholera detection. We followed this population for four-years, making it one of the longest evaluations of the relationship between household WASH and severe cholera incidence, and thereby shedding light on the long-term durability of WASH adaptations. Further, during the development of the composite WASH rule we reduced bias by

For peer review only

categorizing WASH variables without any prior knowledge of cholera incidence rate associated with each component category. We also rigorously validated the WASH prediction rule by using a validation sub-population to ensure that the rule was not over-fitted to the training sub-population.

Our analysis also has limitations. Firstly, household WASH variables were only evaluated once and applied to the whole study period. As household WASH variables were more likely to have improved rather than regressed over time due to the overall secular improvement in socioeconomic conditions in Bangladesh, this misclassification, which would have affected both households classified at baseline as having "Better" WASH and those classified as having "Not Better" WASH, would be expected to have led to more conservative estimates of protective associations with baseline WASH. Secondly, it should be noted that the household WASH variables included in this study were collected in the context of an OCV trial through a brief questionnaire designed to assess confounding variables in the evaluation of vaccine effectiveness. This approach may have led to loss of information, making our estimates of the WASH-severe cholera relationship conservative, and may also have identified variables that are "proxies" for actual water-related factors that directly mediate a reduced risk of severe cholera. Consequently, the WASH variables included in the composite rule may best be interpreted as *markers* of these direct mediators. Nonetheless, because these variables predicted severe cholera risk independently of non-WASH socioeconomic factors, the analysis underscores the importance of water-related WASH adaptations in determining the risk of severe cholera in this setting. Finally, it might be queried whether the relationship between "Better WASH" in the household and a lower risk of severe cholera might reflect the possibility that the "Better WASH" households instituted home-based and clinic-based care for cholera early, and thereby forestalled progression to severe cholera. We think that this was an unlikely explanation for our findings because the study population was highly sensitized to home- and clinic-based early treatment of watery diarrhea, the population had very good access to clinics and hospitals in the proximities of their homes, and all care for diarrhea at these facilities was low cost or free of charge. Moreover, in analyses to be published elsewhere, we found that the household WASH

For peer review only

prediction rule that we developed for severe cholera also strongly predicted the risk of all episodes of cholera seen at the treatment centers, regardless of severity.

Our findings indicate that there are existing culturally acceptable WASH improvements that may be impactful in controlling severe cholera in Mirpur, a dense slum population considered to have hyperendemic cholera. Past experiences in WASH intervention programs have shown that achieving sustainable and effective WASH interventions can be challenging. For example, an evaluation of a household water treatment and handwashing campaign in rural Guatemala found that three years after the intervention there were no differences in handwashing behavior, WASH conditions, or prevalence of childhood diarrhea in the community[18]. Other examples of poor long-term uptake and acceptability of WASH interventions are found in programs implemented in India, Zambia, and Kenya—all highlighting the difficulty of sustained behavior adoption.[19–21] In light of this, understanding *existing* "Better" WASH households and behaviors in endemic slum communities can provide valuable lessons on designing feasible and sustainable WASH interventions.

Recent studies have also found that Mirpur, Bangladesh, the site for this study, has very high rates of microbiological proliferation and contamination in the municipal water supply,[22] likely due to antiquated and now porous water and sewage pipes, making achievable WASH improvements paramount to improving community health. Our analysis found that there *are* existing household water adaptations in Mirpur associated with significantly lowered severe cholera risk, despite contaminated municipal water. The fact that the WASH adaptations practiced by 30% of the population were significantly predictive of lower severe cholera risk even within slum conditions speaks to the potential for existing knowledge to inform cholera control strategies.

Cholera transmission in urban slums may only intensify with the pressures of rapid urbanization combined with climate change effects. Endemic cholera in urban areas is a reality in Bangladesh that faces challenges that are different from previously studied epidemic and rural cholera transmission. Closely examining how some urban households have already made WASH

For peer review only - http://bmjopen.bmj.com/site/about/guidelines.xhtml

adaptations to reduce cholera risk may help design effective cholera control programs that are sustainable and achievable in similar settings.

Data Availability Statement

Data are currently available upon reasonable request and will soon be made available in a public, open access data repository.

Ethics Statement

This study is a re-analysis of data from a cluster randomized trial entitled 'Introduction of cholera vaccine in Bangladesh (ICVB)' conducted by the icddr,b in 2011. The ICVB trial received Institutional Review Board approval (icddr,b protocol number PR#17124) and Bangladesh National Research Ethics Committee approval. Participants gave informed consent to participate in the study before taking part.

Author Contributions

JDC conceptualized the study. SK and FC prepared the original draft. Data analysis was carried out by JP, FA, and DRK. Additionally, JDC, FQ, FM, JHK, and AIK supervised the study. TA, BTT, TI, JI, ABA, MH, GD, FK, XL, and KZ participated in interpreting the data. All authors reviewed and approved the final manuscript.

Competing Interests

There are no competing interests from any of the authors.

Funding

This work was supported, in whole or in part, by the Bill & Melinda Gates Foundation [INV 025386]. Under the grant conditions of the Foundation, a Creative Commons Attribution 4.0 Generic License has already been assigned to the Author Accepted Manuscript version that might arise from this submission.

Acknowledgement

For peer review only

Icddr,b is grateful to the Governments of Bangladesh, Canada, Sweden and the UK for providing unrestricted support. Thanks to Dr. Nigel McMillan (Griffith University) for his scientific and technical support in the manuscript. The funders had no role in study design, data collection and analysis, decision to publish, or preparation of the manuscript.

For peer review only

References

1. Ritchie, H. & Roser, M. Urbanization. *Our World Data* (2018).

2. Dhaka: fastest growing megacity in the world. *The World from PRX* https://www.pri.org/stories/2010-09-08/dhaka-fastest-growing-megacity-world.

3. Ross, A. G. P., Zaman, K. & Clemens, J. D. Health Concerns in Urban Slums: A Glimpse of Things to Come? *JAMA* **321**, 1973–1974 (2019).

4. Islam, M. T., Clemens, J. D. & Qadri, F. Cholera Control and Prevention in Bangladesh: An Evaluation of the Situation and Solutions. *J. Infect. Dis.* **218**, S171–S172 (2018).

5. Longini, I. M., Jr. *et al.* Epidemic and Endemic Cholera Trends over a 33-Year Period in Bangladesh. *J. Infect. Dis.* **186**, 246–251 (2002).

6. Asadgol, Z. *et al.* How climate change can affect cholera incidence and prevalence? A systematic review. *Environ. Sci. Pollut. Res.* **27**, 34906–34926 (2020).

7. Salerno, F., Gaetano, V. & Gianni, T. Urbanization and climate change impacts on surface water quality: Enhancing the resilience by reducing impervious surfaces. *Water Res.* **144**, 491–502 (2018).

8. Kumar, M., Deka, J. P. & Kumari, O. Development of Water Resilience Strategies in the context of climate change, and rapid urbanization: A discussion on vulnerability mitigation. *Groundw. Sustain. Dev.* **10**, 100308 (2020).

9. Taylor, D. L., Kahawita, T. M., Cairncross, S. & Ensink, J. H. J. The Impact of Water, Sanitation and Hygiene Interventions to Control Cholera: A Systematic Review. *PLOS ONE* **10**, e0135676 (2015).

10. George, C. M. *et al.* Randomized Controlled Trial of Hospital-Based Hygiene and Water Treatment Intervention (CHoBI7) to Reduce Cholera. *Emerg. Infect. Dis.* **22**, 233–241 (2016).

For peer review only

11. Luby, S. P., Davis, J., Brown, R. R., Gorelick, S. M. & Wong, T. H. F. Broad approaches to cholera control in Asia: Water, sanitation and handwashing. *Vaccine* **38 Suppl 1**, A110–A117 (2020).

12. Childs, L. *et al.* Evaluation of Knowledge and Practices Regarding Cholera, Water Treatment, Hygiene, and Sanitation Before and After an Oral Cholera Vaccination Campaign-Haiti, 2013-2014. *Am. J. Trop. Med. Hyg.* **95**, 1305–1313 (2016).

13. Qadri, F. *et al.* Feasibility and effectiveness of oral cholera vaccine in an urban endemic setting in Bangladesh: a cluster randomised open-label trial. *The Lancet* **386**, 1362–1371 (2015).

14. Strobl, C., Malley, J. & Tutz, G. An Introduction to Recursive Partitioning: Rationale, Application and Characteristics of Classification and Regression Trees, Bagging and Random Forests. *Psychol. Methods* **14**, 323–348 (2009).

15. Hand, D. J. Measuring classifier performance: a coherent alternative to the area under the ROC curve. *Mach. Learn.* **77**, 103–123 (2009).

16. Faraggi, D. & Reiser, B. Estimation of the area under the ROC curve. *Stat. Med.* **21**, 3093–3106 (2002).

For peer review only

For peer review only - http://bmjopen.bmj.com/site/about/guidelines.xhtml

17. Wolfe, M., Kaur, M., Yates, T., Woodin, M. & Lantagne, D. A Systematic Review and Meta-Analysis of the Association between Water, Sanitation, and Hygiene Exposures and Cholera in Case–Control Studies. *Am. J. Trop. Med. Hyg.* **99**, 534–545 (2018).

18. Arnold, B., Arana, B., Mäusezahl, D., Hubbard, A. & Colford, J. M., Jr. Evaluation of a pre-existing, 3-year household water treatment and handwashing intervention in rural Guatemala. *Int. J. Epidemiol.* **38**, 1651–1661 (2009).

19. Makutsa, P. *et al.* Challenges in Implementing a Point-of-Use Water Quality Intervention in Rural Kenya. *Am. J. Public Health* **91**, 1571–1573 (2001).

20. Firth, J. *et al.* Point-of-Use Interventions to Decrease Contamination of Drinking Water: A Randomized, Controlled Pilot Study on Efficacy, Effectiveness, and Acceptability of Closed Containers, Moringa oleifera, and In-home Chlorination in Rural South India. *Am. J. Trop. Med. Hyg.* **82**, 759–765 (2010).

21. Peletz, R. *et al.* Follow-Up Study to Assess the Use and Performance of Household Filters in Zambia. *Am. J. Trop. Med. Hyg.* **89**, 1190–1194 (2013).

For peer review only

22. Acharjee, M., Rahman, F., Jahan, F. & Noor, R. Bacterial Proliferation in Municipal Water Supplied in Mirpur Locality of Dhaka City, Bangladesh: Microbiological Study of Municipal Water. *CLEAN - Soil Air Water* **42**, 434–441 (2014).

For peer review only

Figure Legends

Figure 1. Map of ICVB study area and cholera surveillance treatment centers

Figure 2. CONSORT- Dynamic population during 4-year follow up period

Figure 3. Decision rule predicting severe cholera episode risk in the training subpopulation

Figure 4. ROC curve for the performance of the decision rule in the training subpopulation

Table 1. Baseline characteristics of the total, training, and validation subpopulations

	Total Population	Training sub-population	Validation sub-population
	n=193576	n=96943	n=96633
Age in years - mean (std)	22.9 (15.4)	22.9 (15.3)	22.9 (15.4)
Gender: Male - n (%)	94008 (48.6)	47365 (48.9)	46643 (48.3)
Monthly Expenditure† – mean (std)	10288.6 (5374.1)	10293.1 (4891.7)	10284.1 (5817.7)
Ownership: Own house - n (%)	28677 (14.8)	14549 (15.0)	14128 (14.6)
House: having one room - n (%)	165215 (85.3)	82427 (85.0)	82788 (85.7)
Wall: Brick/Cement - n (%)	139860 (72.3)	69880 (72.1)	69980 (72.4)

** std: standard deviation; n: number of individuals*
† expenditure in Bangladeshi takas (BDT)

Table 2: Bivariate relationship of WASH variables with severe cholera risk in the training subpopulation

**WASH Variable** *Criteria for Better WASH categorization*	Better WASH				Not Better WASH				Protective association between variable and severe cholera	
	n	Cases	Person-years	IR (95% CI) Per 100000/PY	n	Cases	Person-years	IR (95% CI) Per 100000/PY	Crude (95%CI)	*p*-value
**Shared toilet** *No*	4620	9	10253	88 (46, 169)	92323	135	134076	101 (85, 119)	21 (-55, 60)	0.49
**Toilet type** *Sanitary latrine with or without flush*	80974	122	118747	103 (86, 123)	15969	22	25582	86 (57, 131)	-24 (-95, 21)	0.36
**Drinking Water Source:** *Own tap/well/pump/bottled or vendor water*	4187	5	8993	56 (23, 134)	92756	139	135337	103 (87, 121)	51 (-20, 80)	0.12
**Water availability:** *Tap/tube well/well water is available all the time*	67831	95	93293	102 (83, 125)	29112	49	51036	96 (73, 127)	-15 (-64, 19)	0.43
**Distance to source of drinking water** *Using median as cut off*	43806	47	64461	73 (55, 97)	53137	97	79869	121 (100, 148)	39 (14, 57)	<0.01

For peer review only - http://bmjopen.bmj.com/site/about/guidelines.xhtml

For peer review only

**Drinking Water Treatment:** *Filtered/Boiled/chemical treated*	54275	60	77380	78 (60, 100)	42668	84	66950	125 (101, 155)	36 (11, 54)	<0.01
**Waste disposal location**: *Fixed*	80198	109	116056	94 (78, 113)	16745	35	28273	124 (89, 172)	20 (-17, 46)	0.25
**Hand Washing Water Available** § *Yes*	93758	138	138159	100 (85, 118)	3185	6	6170	97 (44, 216)	-11 (-152, 51)	0.8
**Hand Washing Soap Available** § *Yes*	91514	133	135316	98 (83, 117)	5429	11	9014	122 (68, 220)	17 (-54, 55)	0.56
**Shared kitchen** *No*	86713	118	120182	98 (82, 118)	10230	26	24147	108 (73, 158)	-5 (-62, 32)	0.83

** IR: incidence rate; n: number of individuals*

§ Indicates WASH condition observed directly by study team

For peer review only

Table 3. Protective association between "Better" WASH and severe cholera risk in the total study population

Age at start of follow-up	"Better" WASH				"Not Better" WASH				Protective association between "Better" WASH and severe cholera			
	n	Cases	Person-years	IR per 100000/PYs (95% CI)	N	Cases	Person-years	IR per 100000/PYs (95% CI)	Crude (95%CI)	*p*-value	Adjusted* (95%CI)	*p*-value
< 5 years	7552	5	10518	48 (20, 114)	17149	42	24366	172 (127, 233)	72 (25, 89)	0.012	69 (15, 88)	0.022
5-14 years	8861	4	14618	27 (10, 73)	25318	32	42389	75 (53, 107)	63 (6, 85)	0.037	63 (6, 85)	0.037
15+ years	40983	39	59680	65 (48, 89)	93713	170	136945	124 (107, 144)	46 (20, 64)	0.002	43 (16, 61)	0.004
All	57396	48	84817	57 (43, 75)	136180	244	203701	120 (106, 136)	52 (34, 65)	<0.001	47 (29, 61)	<0.001

** IR: incidence rate; n: number of individuals*

** Adjusted for design effect and selected covariates by stepwise selection using cutoff 0.1 for both of elimination and addition; For entire population, selected covariates were age, gender, home ownership and house having one room; For <5 years, selected covariate was home ownership; For 5-14 years, no covariate was selected; For 15+ years, selected covariates were age, gender and house having one room*

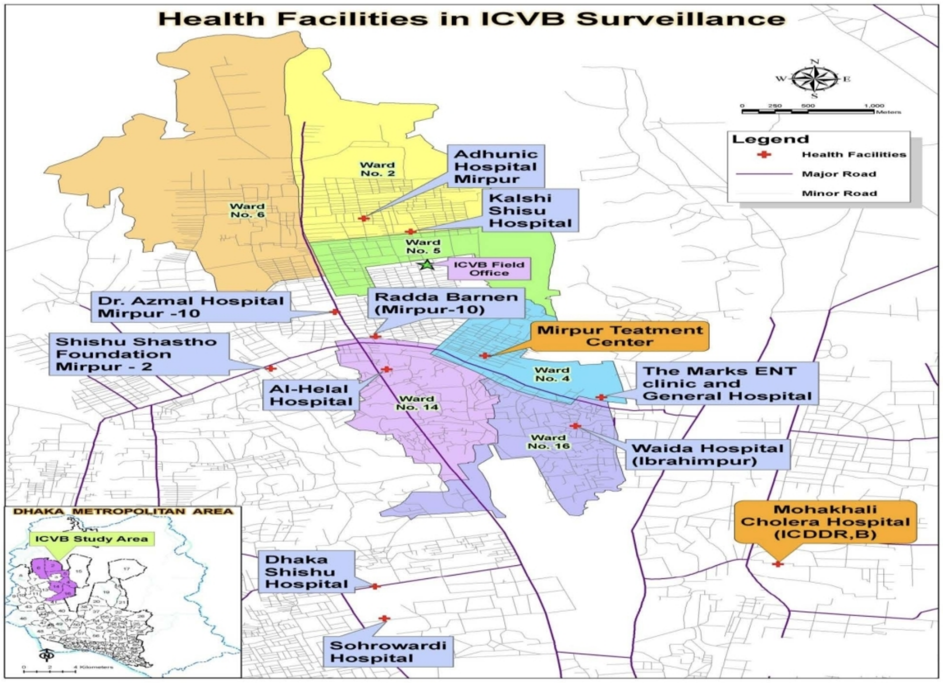

Figure 1. Map of ICVB study area and cholera surveillance treatment centers

221x177mm (237 x 211 DPI)

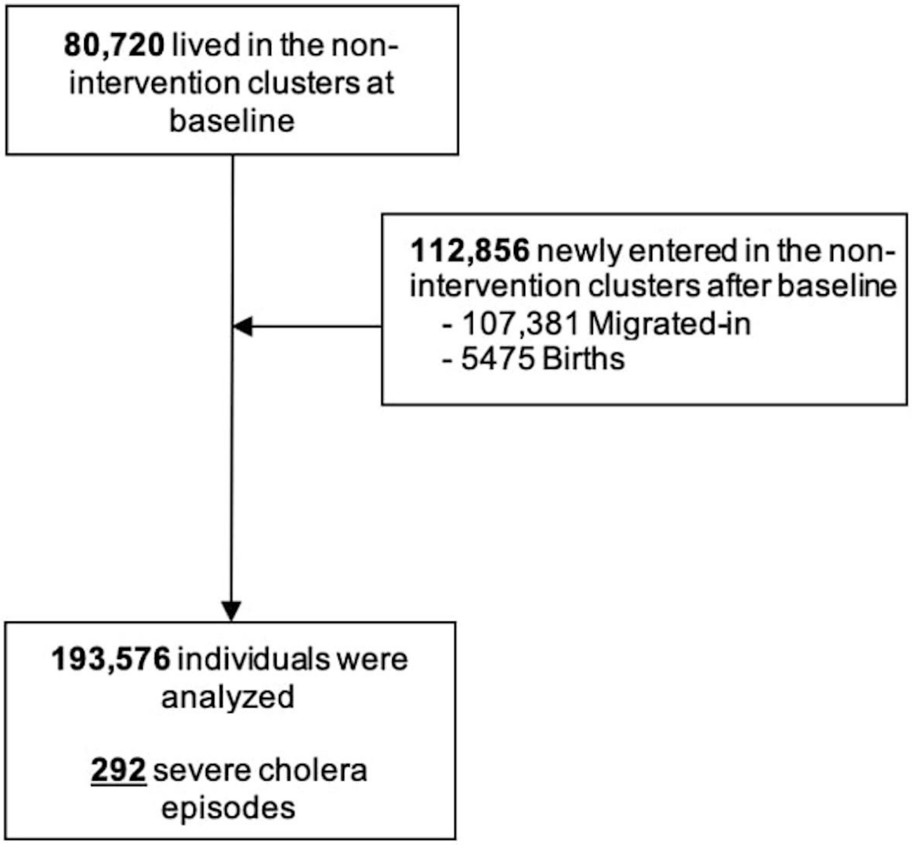

Figure 2: CONSORT- Dynamic population during 4-year follow-up period

109x102mm (300 x 300 DPI)

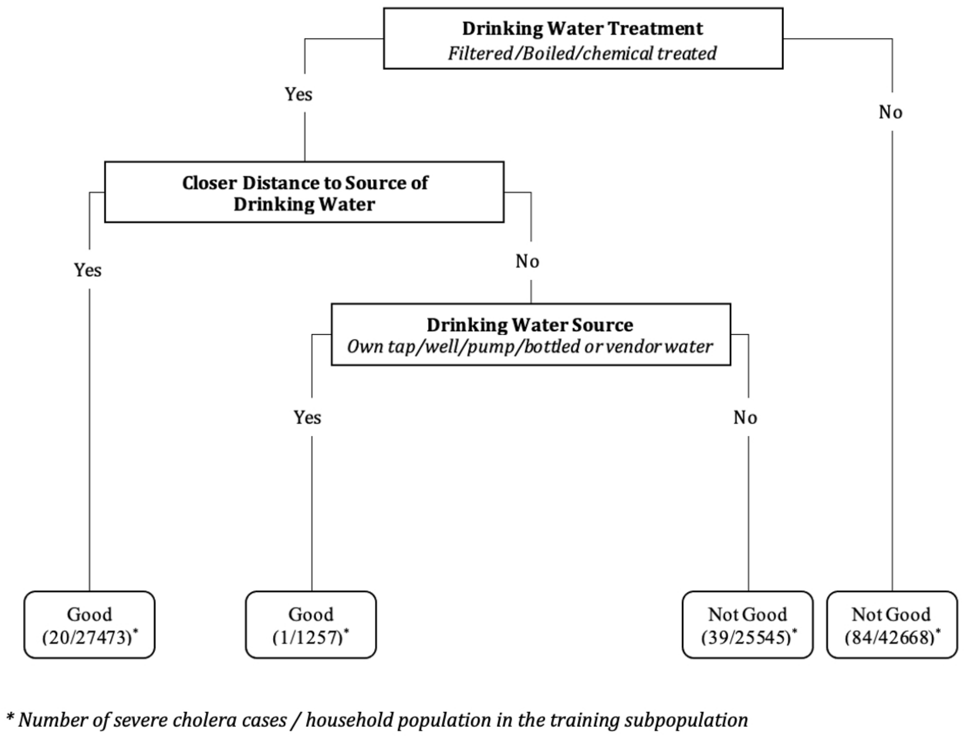

Figure 3. Decision rule predicting severe cholera episode risk in the training subpopulation

202x156mm (144 x 144 DPI)

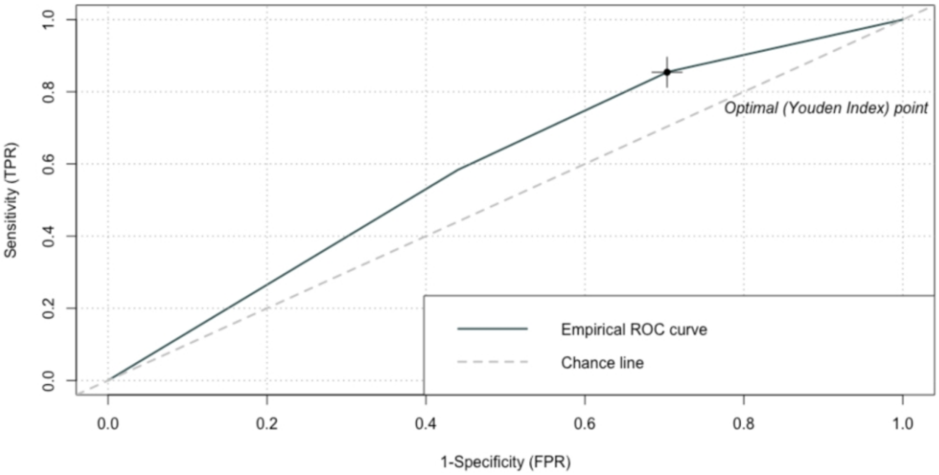

AUC = 59% (95% CI, 55-63); optimal cut-off probability using the Youden index = 0.0012

Figure 4. ROC curve for the performance of the decision rule in the training subpopulation

209x116mm (144 x 144 DPI)

STROBE Statement—Checklist of items that should be included in reports of ***cohort studies***

	Item No	Recommendation	Page No
**Title and abstract**	1	(*a*) Indicate the study's design with a commonly used term in the title or the abstract	1
		(*b*) Provide in the abstract an informative and balanced summary of what was done and what was found	2
**Introduction**			
Background/rationale	2	Explain the scientific background and rationale for the investigation being reported	4
Objectives	3	State specific objectives, including any prespecified hypotheses	4
**Methods**			
Study design	4	Present key elements of study design early in the paper	1-2
Setting	5	Describe the setting, locations, and relevant dates, including periods of recruitment, exposure, follow-up, and data collection	2, 4-5
Participants	6	(*a*) Give the eligibility criteria, and the sources and methods of selection of participants. Describe methods of follow-up	5-6
		(*b*) For matched studies, give matching criteria and number of exposed and unexposed	N/A
Variables	7	Clearly define all outcomes, exposures, predictors, potential confounders, and effect modifiers. Give diagnostic criteria, if applicable	5-6
Data sources/ measurement	8*	For each variable of interest, give sources of data and details of methods of assessment (measurement). Describe comparability of assessment methods if there is more than one group	5-7
Bias	9	Describe any efforts to address potential sources of bias	6-7
Study size	10	Explain how the study size was arrived at	N/A
Quantitative variables	11	Explain how quantitative variables were handled in the analyses. If applicable, describe which groupings were chosen and why	6-7
Statistical methods	12	(*a*) Describe all statistical methods, including those used to control for confounding	6-8
		(*b*) Describe any methods used to examine subgroups and interactions	6-8
		(*c*) Explain how missing data were addressed	N/A
		(*d*) If applicable, explain how loss to follow-up was addressed	6
		(*e*) Describe any sensitivity analyses	N/A
**Results**			
Participants	13*	(a) Report numbers of individuals at each stage of study—eg numbers potentially eligible, examined for eligibility, confirmed eligible, included in the study, completing follow-up, and analysed	8
		(b) Give reasons for non-participation at each stage	N/A
		(c) Consider use of a flow diagram	12
Descriptive data	14*	(a) Give characteristics of study participants (eg demographic, clinical, social) and information on exposures and potential confounders	13
		(b) Indicate number of participants with missing data for each variable of interest	N/A
		(c) Summarise follow-up time (eg, average and total amount)	8
Outcome data	15*	Report numbers of outcome events or summary measures over time	8

For peer review only

Main results	16	(*a*) Give unadjusted estimates and, if applicable, confounder-adjusted estimates and their precision (eg, 95% confidence interval). Make clear which confounders were adjusted for and why they were included	9
		(*b*) Report category boundaries when continuous variables were categorized	N/A
		(*c*) If relevant, consider translating estimates of relative risk into absolute risk for a meaningful time period	N/A
Other analyses	17	Report other analyses done—eg analyses of subgroups and interactions, and sensitivity analyses	11
**Discussion**			
Key results	18	Summarise key results with reference to study objectives	10
Limitations	19	Discuss limitations of the study, taking into account sources of potential bias or imprecision. Discuss both direction and magnitude of any potential bias	12
Interpretation	20	Give a cautious overall interpretation of results considering objectives, limitations, multiplicity of analyses, results from similar studies, and other relevant evidence	11-13
Generalisability	21	Discuss the generalisability (external validity) of the study results	12-13
**Other information**			
Funding	22	Give the source of funding and the role of the funders for the present study and, if applicable, for the original study on which the present article is based	13

*Give information separately for exposed and unexposed groups.

Note: An Explanation and Elaboration article discusses each checklist item and gives methodological background and published examples of transparent reporting. The STROBE checklist is best used in conjunction with this article (freely available on the Web sites of PLoS Medicine at http://www.plosmedicine.org/, Annals of Internal Medicine at http://www.annals.org/, and Epidemiology at http://www.epidem.com/). Information on the STROBE Initiative is available at http://www.strobe-statement.org.

For peer review only
